# Vascular Endothelial Growth Factor Augments the Tolerance Towards Cerebral Stroke by Enhancing Neurovascular Repair Mechanism

**DOI:** 10.1007/s12975-022-00991-z

**Published:** 2022-02-17

**Authors:** Adnan Ghori, Vincent Prinz, Melina Nieminen-Kehlä, Simon. H. Bayerl, Irina Kremenetskaia, Jana Riecke, Hanna Krechel, Thomas Broggini, Lea Scherschinski, Tamar Licht, Eli Keshet, Peter Vajkoczy

**Affiliations:** 1 Department of Neurosurgery, Universitätsmedizin Charité, 10117 Berlin, Germany; 2 Department of Developmental Biology and Cancer Research, Hebrew University Hadassah Medical School, 91120 Jerusalem, Israel

**Keywords:** Blood–brain barrier, Ischemic stroke, Angiogenesis, Vascular biology

## Abstract

The breakdown of the blood–brain barrier (BBB) is a critical event in the development of secondary brain injury after stroke. Among the cellular hallmarks in the acute phase after stroke are a downregulation of tight-junction molecules and the loss of microvascular pericyte coverage and endothelial sealing. Thus, a rapid repair of blood vessel integrity and re-stabilization of the BBB is considered an important strategy to reduce secondary brain damage. However, the mechanisms underlying BBB disruption remain poorly understood. Especially, the role of VEGF in this context remains inconclusive. With the conditional and reversible VEGF expression systems, we studied the time windows of deleterious and beneficial VEGF actions on blood vessel integrity in mice. Using genetic systems for gain of function and loss of function experiments, we activated and inhibited VEGF signaling prior and simultaneously to ischemic stroke onset. In both scenarios, VEGF seems to play a vital role in containing the stroke-induced damage after cerebral ischemia. We report that the transgenic overexpression of VEGF (GOF) prior to the stroke stabilizes the vasculature and prevents blood–brain barrier disruption in young and aged animals after stroke. Whereas inhibition of signals for endogenous VEGF (LOF) prior to stroke results in bigger infarction with massive brain swelling and enhanced BBB permeability, furthermore, activating or blocking VEGF signaling after ischemic stroke onset had comparable effects on BBB repair and cerebral edema. VEGF can function as an anti-permeability factor, and a VEGF-based therapy in the context of stroke prevention and recovery has an enormous potential.

**Supplementary Information:**

The online version contains supplementary material available at 10.1007/s12975-022-00991-z.

## Introduction

Cerebrovascular diseases include 50% of all the neurological diseases in western countries and are the second leading cause of death after cardiovascular diseases worldwide [1]. A major complication following stroke is the disruption of the blood–brain barrier (BBB) and development of brain swelling, secondarily aggravating brain injury. Under the normal condition, BBB is intact and stable among other due to pericytes and tight junction proteins (TJPs). However, under the pathological conditions, such as stroke, the BBB is temporarily disrupted, pericyte coverage is reduced, and TJPs expression is diminished [2]. The accompanied tissue edema and the activation of inflammatory processes play an important role in aggravating secondary brain injury. Vascular endothelial growth factor (VEGF) is a growth factor playing various roles in vasculogenesis and angiogenesis during development and is likewise essential in maintaining postnatal vascularization [3–5]. In addition, VEGF in its capacity as a hypoxia-induced angiogenic factor is thought to play a pivotal role in post-infarct neovascularization [6]. VEGF being a potent angiogenic trigger has been discussed to improve outcome after stroke [7–9]. Unfortunately, VEGF is also thought to function as a vascular permeability factor. One general notion is that stroke-related hypoxia leads to an upregulation of VEGF which in turn is responsible for the vascular edema and blood–brain barrier breakdown in the subacute period, i.e., 72 h after stroke [10, 11]. Because of the profound effects VEGF has on blood vessels, its potential therapeutic use has been explored in oncology and cardiovascular medicine [12–14]. Bevacizumab, a potent VEGF signaling inhibition, is used in various types of cancers and AMD [15, 16]. On the contrary, high-dose VEGF treatment showed a significant improvement in patients after chronic myocardial ischemia [17]. Pursuing VEGF as a therapeutic agent after stroke is a promising clinical strategy; however, it still lacks convincing efficacy [18–20]. Many, potential attempts to use VEGF-induced therapies have repeatedly failed due to the lack of adequate models. To optimize the beneficial actions of VEGF following cerebral ischemia, it is necessary to focus on the correct timing of VEGF administration [21–24]. We hypothesize that VEGF in its capacity as a hypoxia-induced angiogenic factor can play a pivotal role in reducing brain swelling and enhancing post-stroke recovery.

Here, we took advantage of a neuron-specific promotor and manipulated VEGF expression conditionally in the brain to understand the time windows of VEGF action on the blood–brain barrier and vascular edema. Using VEGF gain-of-function system (GOF) we were able to demonstrate that VEGF reduces brain edema by increasing vascular stability and reduces infarct size after ischemic stroke. In contrast, blocking VEGF signaling using a loss-of-function system (LOF) led to disruption of the BBB and vascular integrity resulting in massive brain swelling after ischemic stroke. Our experiments redefine the role of VEGF as well as its therapeutic potential in ischemic cerebral stroke.

## Material and Methods

Please see the Supplementary Information Materials and Methods sections for detailed experimental description.

### Animals

Twelve to 14 weeks and 16–20-months-old animals (a tet-responsive VEGF GOF (gain of function) and VEGF LOF (loss of function). Both genders are used for this study to exclude any gender related differences. The average weight is 26.32 g. Average age for young animals was 13.04 weeks and for aged animal 17.31 months. For switching-off of VEGF or I-sVEGF-R1, water was supplemented by 500 mg/L tetracycline (Tevacycline; Teva) and 3% sucrose. For switching on the transgenes, tetracycline-supplemented water was replaced by fresh water for the desired time. The animals were maintained at 22 °C room with a 12-h light/dark cycle and received drinking water ad libitum.

### Model of Cerebral Ischemia

Animals were anesthetized with a combination of ketamin and rompun injection. Animals were placed in supine position, and the skin of the ventral neck was disinfected. Middle cerebral artery occlusion (MCAO) was performed as described [25, 26]. A midline neck incision is made, and the soft tissues are pulled apart. The common carotid artery (CCA) is carefully dissected free from the surrounding nerves (without harming the vagus nerve), and a ligature is made using suture thread. A small hole is cut in the CCA before it bifurcates to the (external carotid artery) ECA and the (internal carotid artery) ICA. Cerebral infarcts were produced by 60-min left MCA occlusion followed by reperfusion. Occlusion was conducted by introducing a 7–0-intraluminal suture (Doccol Corporation) in the internal carotid artery and advanced it. After 60 min, the filament was withdrawn to allow reperfusion. The temperature was obtained at 37 °C with a temperature control table (Medax, Germany).

### Evans Blue Administration and Quantification

The integrity of blood–brain barrier was assessed by administration of Evans Blue dye. A 2% Evans Blue solution in saline was administered intravenously at a volume of 100 mg/kg at 24 h, 72 h, and 7-day post-stroke. Evans Blue was always administered 4 h prior to animal sacrifice. Animals were perfused with PBS; brains were frozen and processed for the presence of Evans Blue using fluorescence microscope. The integrity of blood–brain barrier was assessed by administration of Evans Blue dye. For the quantification of the extravasation area, the Evans Blue accumulation area on a 200 × field (i.e., 20 × objective lens and 10 × ocular; 0.7386 mm^2^ per field) was measured and quantified using ImageJ (NIH) software. For the quantification of the leaky vessel, all the vessels (labelled with CD31) which showed Evans Blue deposition or accumulation around them in the 200 × field were considered to be leaky. These vessels were counted and quantified using ImageJ software. All quantifications were done blinded to the researcher.

### Quantification of Angiogenesis and Vessel Counting

Blood vessel counting followed procedures as previously published [27, 28]. For quantification of angiogenesis, the numbers of branch points were counted; the number of blood vessel branch points is indicative of the number of new blood vessel sprouts arising from pre-existing blood vessels. Vessel density was determined from infarct core, peri-infarct, and contralateral area on a 200 × field (i.e., 20 × objective lens and 10 × ocular; 0.7386 mm^2^ per field). ImageJ (NIH) software was used for quantification. Quantification was done blinded.

### Neurological Score

A global neurological assessment, the Bederson test, was conducted to measure neurological impairments after stroke [29] (see Supplementary materials and methods for detailed protocol).

### Statistical Analysis

Results are presented as mean ± SEM. Data was analyzed using statistical program Graphpad Prism. Statistical significance was determined by Student’s *t*-test and ANOVA when indicated. Mann–Whitney test was used for Bederson test. *P < 0,05, **P < 0.01, and ***P < 0.001 were considered statistically significant.

## Results

### Conditional CNS-Specific Stimulation and Inhibition of VEGF Signaling

A well-established tetracycline-regulated system was used to study VEGF-specific changes after cerebral ischemia. A conditional and reversible VEGF induction was achieved by calmodulin kinase IIα (CamKIIα) promoter-driven transactivator protein. For VEGF-GOF (gain-of-function), a tet-responsive VEGFA164 responder line was used and for VEGF LOF (loss-of-function), a transgene responder encoding a chimeric tet-regulated protein composed of the five Ig-like loops of the extracellular domain of Flt1 fused to an IgG1-Fc tail was used. The induced secreted receptor (I-sVEGF-R1) efficiently binds and sequesters VEGF, thereby precluding it signaling. Thus, this transgenic mouse system allows reversible induction and repression of VEGF signaling at will (as outlined in previous publication Licht et al. [30]) (Supplementary Fig. 1a). Changes in VEGF expression dynamics pre- and post-transgenic manipulation for GOF and LOF were confirmed prior to the experiments. VEGF mRNA expression was slightly increased in VEGF-GOF animals already 72-h post tetracycline withdrawal, whereas five-fold VEGF upregulation was observed after 28 days (Supplementary Fig. 1b). Relative levels of gene expressions were standardized to control levels. VEGF expression level in VEGF-LOF animals was similar to control animals. Immunostaining of the extracellular domain of FLT1 in CamKIIα -tTa/TET- I-sVEGF-R1 double transgenic animals after 28 days of tetracycline withdrawal confirmed expression of the VEGF-trapping protein in VEGF-LOF animals (Supplementary Fig. 1c).

### Pre-stroke VEGF Activation Reduces Cerebral Edema and Stabilizes the Vascular Integrity After Cerebral Ischemia

**Fig. 1:**
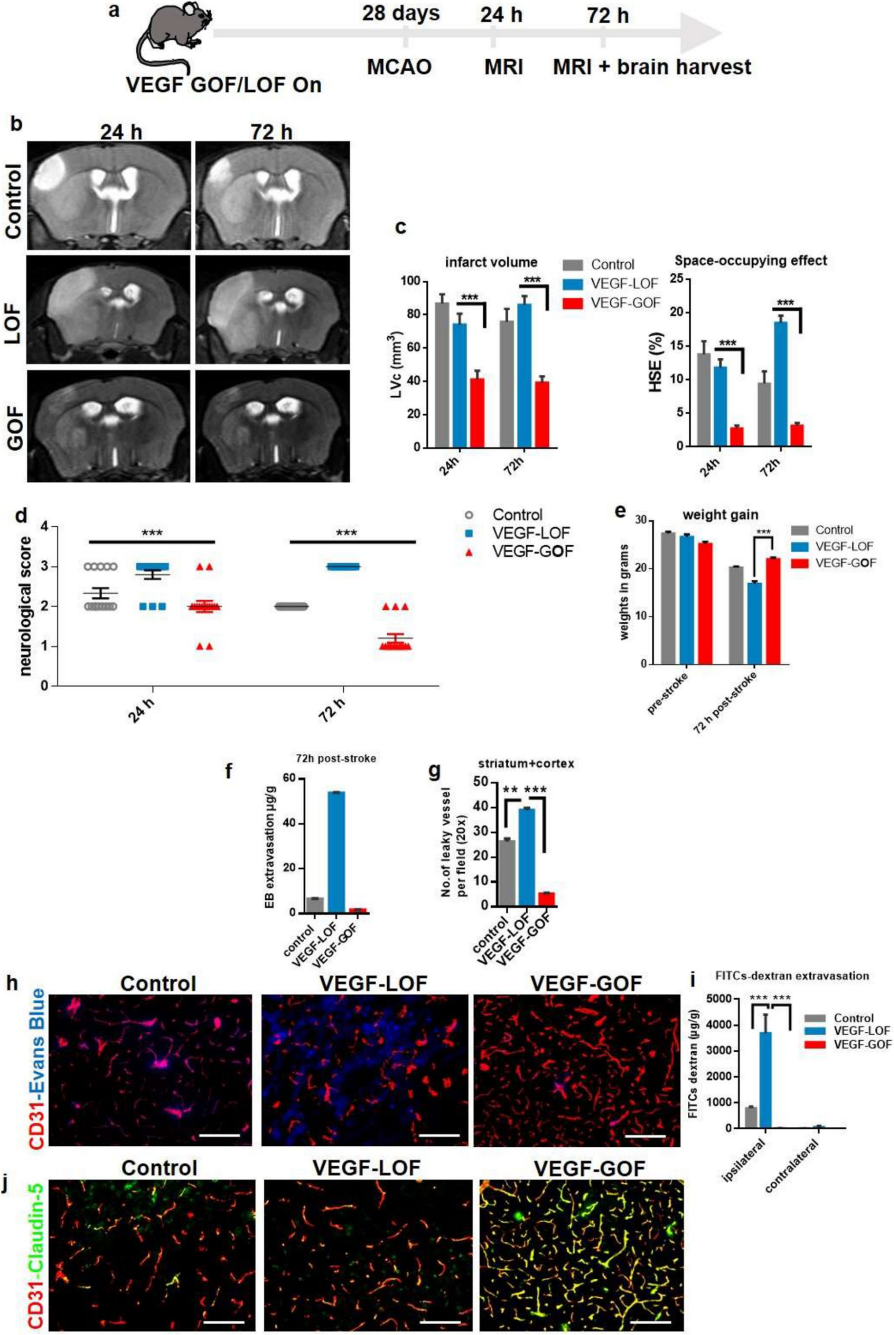
VEGF acts as an anti-permeability factor after cerebral ischemia. **(a)** Schematic timeline representation of the experimental procedure. **(b)** Cerebral lesion region is determined by using T2-weighted magnetic resonance images (MRI), representative MRI from control, VEGF-Loss of Function (LOF) and VEGF-Gain of Function (GOF) animal 24 h and 72 h post-stroke. **(c)** MRI-based quantitation of the lesion volume corrected (LVc, mm3) and space-occupying effect attributable to edema (% HSE) after stroke. Infarct size is significantly reduced in GOF animals already from 24 h post-stroke, whereas LOF show a significant increase in the infarct volume 24 h post-stroke and the infarct size further increase after 72 h post-stroke. The space-occupying effect in VEGF-GOF is significantly reduced 24 h post-stroke indicating a decrease in the volume of brain swelling, on the other hand blocking VEGF signaling (LOF) aggravated brain swelling. *n* = 10 animals per experimental groups. P values were determined by ANOVA (***P < 0,001). Values are represented as ± SEM. **(d)** Neurological function recovers in the VEGF-GOF 72 h post-stroke when compared to the VEGF-LOF and control (***P < 0.001). **(e)** VEGF-LOF animal do not gain weight as fast as VEGF-GOF and control animals 72 h post-stroke. *n* = 10 animals per experimental groups. **(f)** Blood–brain barrier permeability assessed by Evans Blue extravasation (μg/g brain tissue) in control, VEGF-LOF and VEGF-GOF (*n* = 4 per group) at 72 h after 60 min of MCAO. **(g)** Measurement of number of leaky vessels showed a significantly reduced vessel permeability and improved blood–brain barrier function in VEGF-GOF when compared to control and VEGF-LOF. P values were determined by ANOVA (**P < 0.01, ***P < 0.001). **(h)** Immunofluorescence staining reveals a significantly increased in cerebrovascular permeability in the ischemic core and delay of vascular repair in LOF animals versus GOF and control animals (n = 8 in all experimental groups). **(i)** Extravasation experiments with low molecular weight Fluorescein Isothiocyanate (FITC)-Dextran showed increased extravasation in the VEGF-LOF (n = 3 in all experimental groups, ***P < 0.001). **(j)** Tight junction protein claudin-5 showed increased blood vessel coverage in VEGF-GOF, whereas blood vessels in VEGF-LOF and control have lower claudin-5 coverage. Magnification 200 × ; Scale bar 50 μm

We used a 60-min middle cerebral artery occlusion (MCAO) and reperfusion protocol, which leads to a focal, ischemic cell death in striatum and cortex. To achieve high local brain tissue levels of VEGF at the time of stroke onset, tetracycline was withdrawn 28 days prior to filament vessel occlusion (Fig. 1a). In control animals, the 60-min MCAO protocol resulted in hemispheric stroke and significant brain swelling 24 and 72 h after reperfusion as demonstrated by T2-weighted magnetic resonance imaging (Fig. 1b). VEGF-LOF aggravated the size of infarction area with an even larger space-occupying effect due to brain swelling (Fig. 1b, c). VEGF-GOF significantly reduced infarct volume and space-occupying effect due to reduced brain swelling within 72 h after stroke (Fig. 1b, c). In coherence with these results, the Bederson score, a behavior test assessing the neurological function of animals, was significantly improved in the VEGF-GOF animals at 72 h post-stroke, whereas inhibiting the VEGF signal (VEGF-LOF) worsened the neurological status of animals reflected by a low Bederson score and also hindered regaining of the body weight (Fig. 1d, e).

**Fig. 2:**
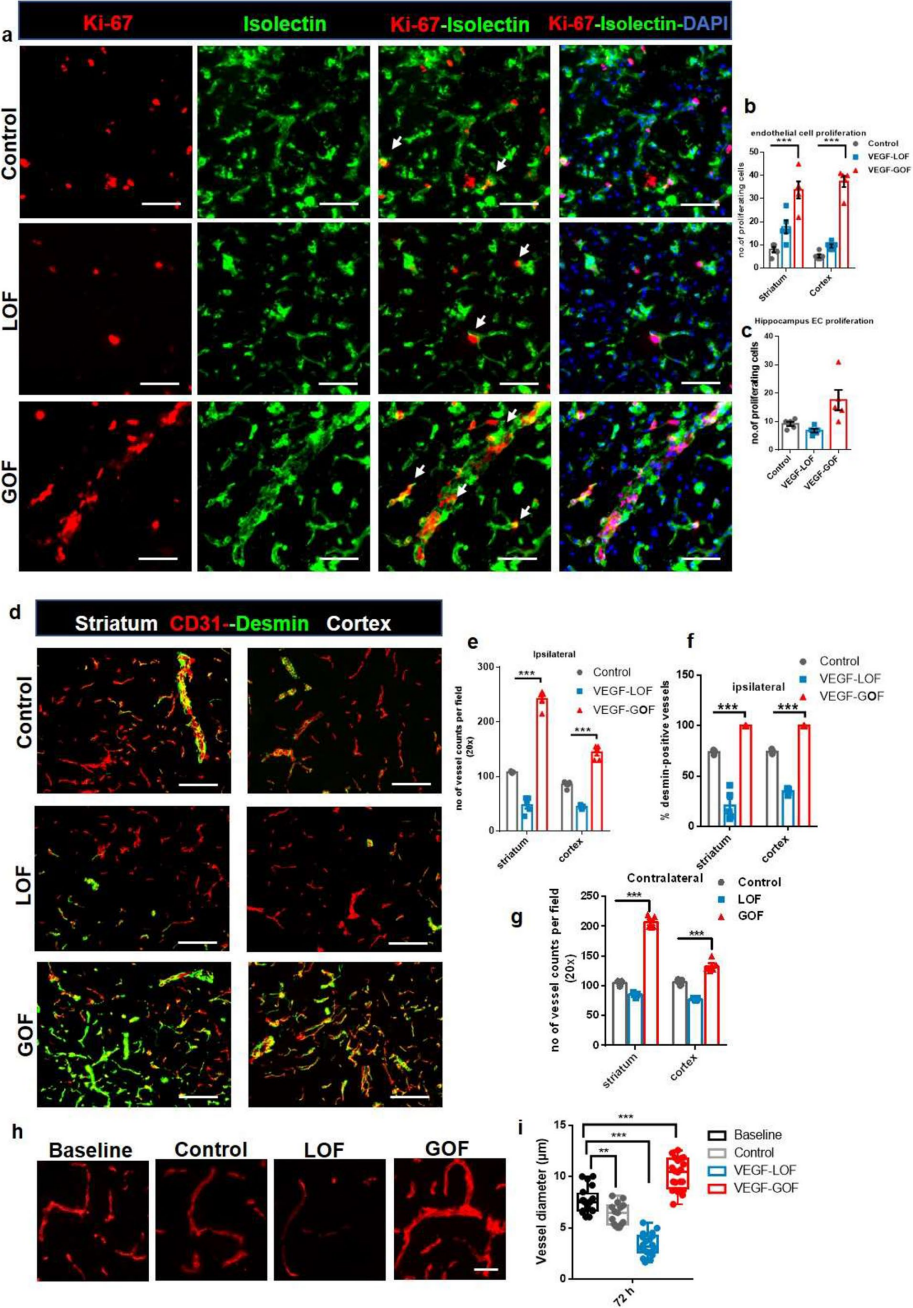
VEGF overexpression accelerated vascular recovery via enhanced endothelial–pericyte interactions and blood vessel sealing. **(a–c)** VEGF overexpression enhances endothelial cell (EC) proliferation. KI-67 labelled cells co-stained with neovascular marker isolectin, showed increased EC proliferation in the VEGF-GOF 72 h post-stroke. On the contrary, VEGF-LOF and control showed fewer proliferating endothelial cells in striatum, cortex, and hippocampus (***P < 0.001). **(d)** CD31/ desmin co-staining on cryofixed sections shows the enhanced pericyte coverage of cerebral vessel in VEGF-GOF animals compared with LOF and control animals. **(e, f)** Total number of vessels were increased in the animals with the VEGF overexpression and quantification of desmin-positive vessels show increase in percentage of stable vessels, that is, desmin-covered vessels in the VEGF-GOF (***P < 0.001). **(g)** VEGF overexpression (GOF) for 28 days lead to an increase in the number of blood vessel in the contralateral hemisphere prior to stroke (***P < 0.001). n = 10 animals per experimental groups. *P* values were determined by ANOVA. Values are represented as ± SEM. Magnification 200 × ; scale bar 50 μm. **(h)** Fluorescent images of vessels stained with CD31 showing the vessel diameter in baseline, control, VEGF-LOF and VEGF-GOF animals, scale bar 15 µm. **(i)** The box and whiskers plot show the blood vessel diameter in each experimental group (**P < 0.01, ***P < 0.001), *P* values were determined by one-way ANOVA

These differences in space-occupying edema formation suggest distinct impairments of BBB and vascular integrity, leading to vasculogenic edema. To confirm this, we performed extravasation studies by IV injection of Evans Blue, which binds to circulating albumin and represents a large molecular tracer. In order to rule out a hyper-permeabilizing action of local VEGF for Evans Blue, we first confirmed that VEGF-GOF did not induce Evans Blue leakage in non-injured brains, compared to VEGF-LOF (Supplementary Fig. 2a). Evans Blue tracer studies following stroke confirmed that VEGF-GOF attenuates vascular permeability. VEGF-LOF resulted in a massive increase in Evans Blue leakage. Quantification analysis of Evans Blue extravasation confirmed the substantial damage. Quantification analysis of leaky vessels confirmed the substantial damage to the vasculature in VEGF-LOF and rescued vascular integrity in the VEGF-GOF animals 72 h after stroke (Fig. 1f-h). To address whether this vessel sealing effect of VEGF-GOF is limited to the large molecular tracer Evans Blue (60 kDa), we additionally performed the same tracer studies with 4 kDa FITC-dextran. Similarly, VEGF-LOF enhanced FITC-Dextran extravasation, whereas VEGF-GOF blocked 4 kDa FITC-Dextran extravasation (Fig. 1i). The cellular basis for blood–brain barrier disruption is loss of integrity of the neurovascular unit and/or reduced expression of endothelial tight junction molecules (baseline expression of claudin-5 is shown in Supplementary. Figure 2b). Multifluorescence immunostainings for the tight junction protein claudin-5 and occludin (in areas of ischemia-induced vascular damage) revealed decreased surface expression of claudin-5 and occludin in VEGF-LOF. In contrast, blood vessels in VEGF-GOF were vastly covered with claudin-5 and occludin (Fig. 1j and Supplementary Fig. 2c).

Taken together, these results suggest that VEGF works as an anti-permeability factor via strengthening aspects of the neurovascular unit (NVI). Blocking the signals for naturally produced VEGF induces severe vascular damage and affects the blood–brain integrity in the acute ischemia phase.

### Pre-stroke VEGF-Induced Angiogenesis and Decreases Structural Damage in the Ischemic Brain

The induction of angiogenesis has been associated with vascular remodeling, tissue survival, and improving collateral circulation after stroke [31–33]. Next, we sought to determine the effect of VEGF pretreatment on angiogenic processes pre- and post-stroke. First, we analyzed the effects of 28 days of VEGF modulation on the naive brain vasculature. VEGF-GOF led to an enhanced proliferation of endothelial cells. VEGF overexpression turned the vascular system into a highly plastic network with continuous angiogenesis in the entire brain (Supplementary Fig. 3). The continuously newly formed blood vessels were fully covered with pericytes, thus making the vasculature fully stable and functional, without affecting vascular permeability for Evans Blue (Supplementary Fig. 3). However, VEGF-LOF did exhibit neither angiogenic activity nor an increase in vessel density as compared to control animals (Supplementary Fig. 3). Thus, we suggest that blocking of VEGF signaling might not influence the vasculature under physiological conditions.

Following these findings, we studied the effects of VEGF on the dynamics of microvasculature after ischemia. Ki-67 and isolectin co-staining showed elevated endothelial turnover in VEGF-GOF compared to control and VEGF-LOF 72 h post-stroke (Fig. 2a). Similar results were observed with Ki-67-CD31 staining (Supplementary Fig. 4). Quantification of endothelial cell (EC) proliferation showed high EC turnover in all regions (striatum, cortex, and hippocampus) mainly affected by ischemia (Fig. 2b, c). As expected, the number of blood vessels was significantly increased in the ipsilateral hemisphere in VEGF overexpression animals (Fig. 2d, e) and desmin + staining demonstrated coverage of microvasculature by pericyte cells in the ipsilateral hemisphere after ischemia (Fig. 2f). (NG2 another pericyte marker also confirmed this data Supplementary Fig. 5). Interestingly, the number of blood vessels in the contralateral hemisphere of the VEGF overexpression animals was significantly increased as well (Fig. 2g). The data demonstrate that VEGF overexpression reconstitutes the functional microvessels following ischemia. In contrast, inhibiting VEGF signaling affects the number of blood vessels in the ipsilateral hemisphere after stroke. The number of vessels was significantly decreased in VEGF-LOF (Fig. 2d, e). Furthermore, endothelial-pericyte interaction in the ipsilateral region was significantly influenced by the VEGF signal blockage. CD31-Desmin co-staining demonstrated a high number of blood vessels without pericyte coverage in the VEGF-LOF animals after stroke. Quantification data showed that only 25–30% of blood vessels were desmin-positive in the ipsilateral region after stroke in VEGF-LOF animals (Fig. 2f). In addition, vessel diameter analysis revealed an increase in blood vessel diameter of VEGF-GOF animals compared to baseline, control and VEGF-LOF (Fig. 2h, i). The diameter of blood vessels in VEGF-LOF was significantly decreased (Fig. 2h, i).

The data show that VEGF plays a central role in vascular repair and reestablishment of a differentiated CNS specific vascular system. Lack of VEGF hampers this process, whereas VEGF overexpression significantly improves the repair process.

### VEGF Activates Molecular Mediators in Angiogenesis and Pericytes Recruitment

**Fig. 3:**
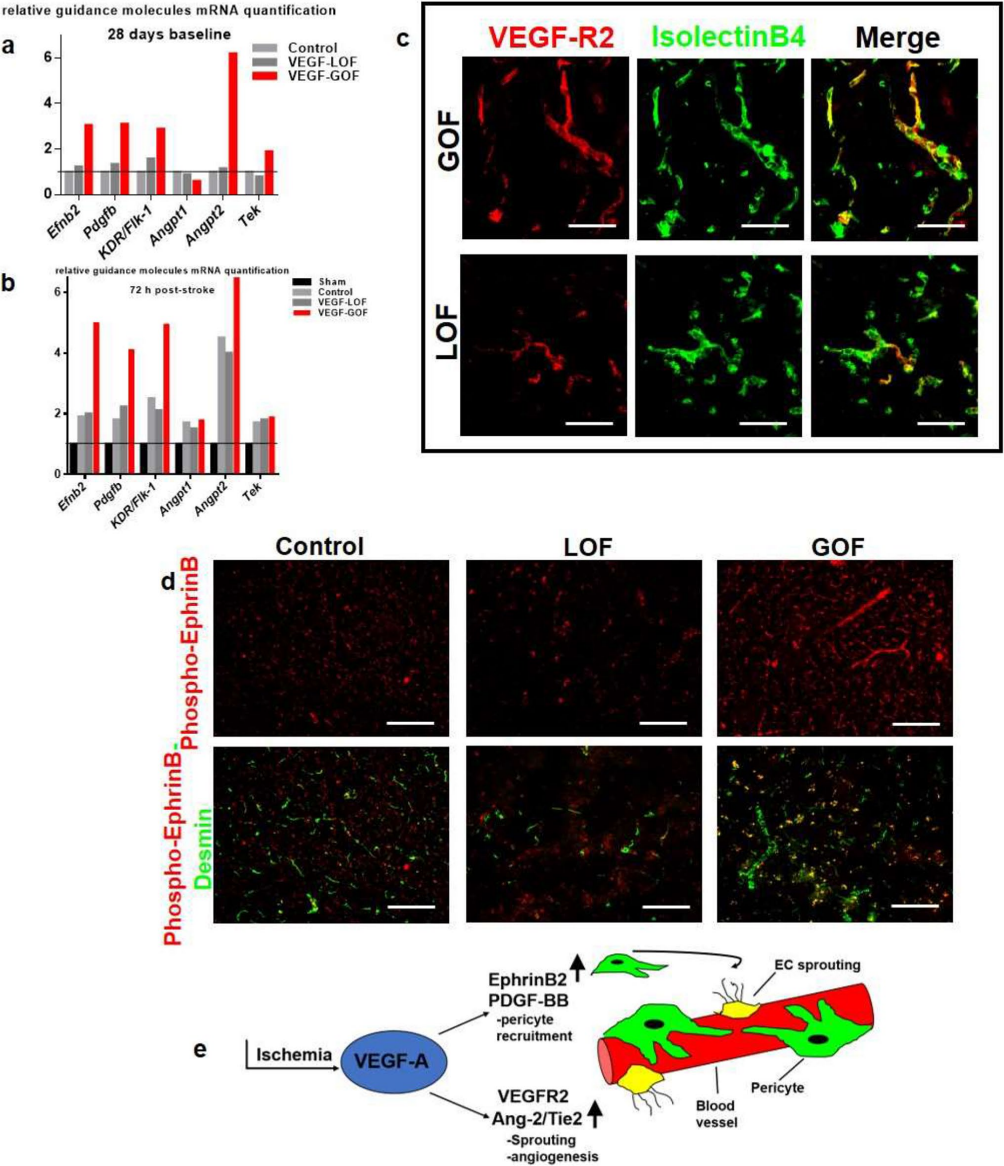
EphrinB2/PDGF-BB and ANG2/Tie2 signaling pathways involved in vascular stability. **(a)** RT-qPCR demonstrates an upregulation of mRNA expression of different guidance molecules ephrinB2, PDGFB, VEGF-R2, Ang-2, and Tie-2 in the VEGF-GOF already under the baseline condition. VEGF-LOF and control showed lower expression level for these molecules (*n* = 4 animals per group). **(b)** Under ischemic condition 72 h after stroke, upregulation was seen in VEGF-GOF, VEGF-LOF and control group, the values were normalized to sham-operated animals. Error bar represent s.e.m. **(c)** Transgenic increase in VEGF-GOF upregulates the VEGFR2 expression and it is colocalized with the blood vessels (labelled with isolectin) 72 h post-stroke (scale bar 20 µm). Blocking VEGF signaling leads to decrease in the VEGFR2 expression. **(d)** increased ephrinB phosphorylation was detected from 72 h in VEGF-GOF compared to VEGF-LOF and control, note the increased ephrinB phosphorylation is mainly detected in pericytes of VEGF-GOF animals. *n* = 5 animals per group. Magnification 200 × ; Scale bar 50 μm. **(e)** Schematic representation of VEGF-induced pathways involved in vascular stability

Our data provide evidence that pre-stroke VEGF activation reduces cerebral edema and augments blood vessels with improved blood–brain barrier integrity within 72 h after ischemia. Subsequently, we aimed to understand the mechanistic pathways triggered by VEGF-GOF when stabilizing the vasculature following ischemia. We conducted real-time qPCR analysis and focused our study on molecules involved in vascular regulatory activities. Under baseline conditions (VEGF activation without stroke), VEGF-GOF showed an upregulation in ephrinB2, VEGFR2, ANG2, and Tie2, as well as downregulation in ANG1. On the other hand, VEGF-LOF did not show any changes in the expression pattern of these vasoactive mediators compared to control animals (Fig. 3a). Nevertheless, this pattern changed following stroke. Stroke-induced upregulation of vasoactive mediators was observed in VEGF-LOF and control groups as well. Both VEGF-LOF and control groups showed two-fold upregulation of ephrinB2, VEGFR2, Tie2, and ANG1, whereas ANG2 was upregulated four-fold compared to control (Fig. 3b). As previously mentioned, VEGF-GOF led to increased levels of vasoactive mediators under physiologic conditions. VEGF-GOF demonstrated expression levels of ephrinB2, VEGFR2, ANG2, and Tie2 that further increased beyond the increased pre-stroke values 72 h after stroke (Fig. 3b). Furthermore, expression patterns of ANG1 in VEGF-GOF were comparably high compared to VEGF-LOF and control after ischemia (Fig. 3b). ANG2 immunofluorescence showed similar expression patterns VEGF-GOF had an increased ANG2 expression under both baseline and ischemic conditions, whereas ANG2 expression in VEGF-LOF was upregulated after ischemia but not under baseline condition (Supplementary Fig. 6a). Immunofluorescence staining confirmed the upregulation of VEGFR2 in endothelial cells of VEGF overexpression groups, whereas VEGF-LOF animals had decreased VEGFR2 expression in EC cells (Fig. 3c).

Angiogenesis has been linked to ephrinB phosphorylation [34]. Therefore, we investigated the status of ephrinB phosphorylation during the angiogenic phase under VEGF manipulation. VEGF-GOF led to increased ephrinB phosphorylation 72 h post-stroke compared to the VEGF inhibition group (Supplementary Fig. 6b). The increase of ephrinB phosphorylation was mainly detected on the perivascular cells as suggested by ephrinB-desmin co-staining (Fig. 3d). Our data suggests that the VEGF overexpression simultaneously activates two diverse pathways to form new blood vessel and to concurrently stabilize the newly formed vasculature (Fig. 3e).

### Pre-stroke VEGF Upregulation Mediates Vessel Integrity and BBB Repair in Aged Animals

**Fig. 4:**
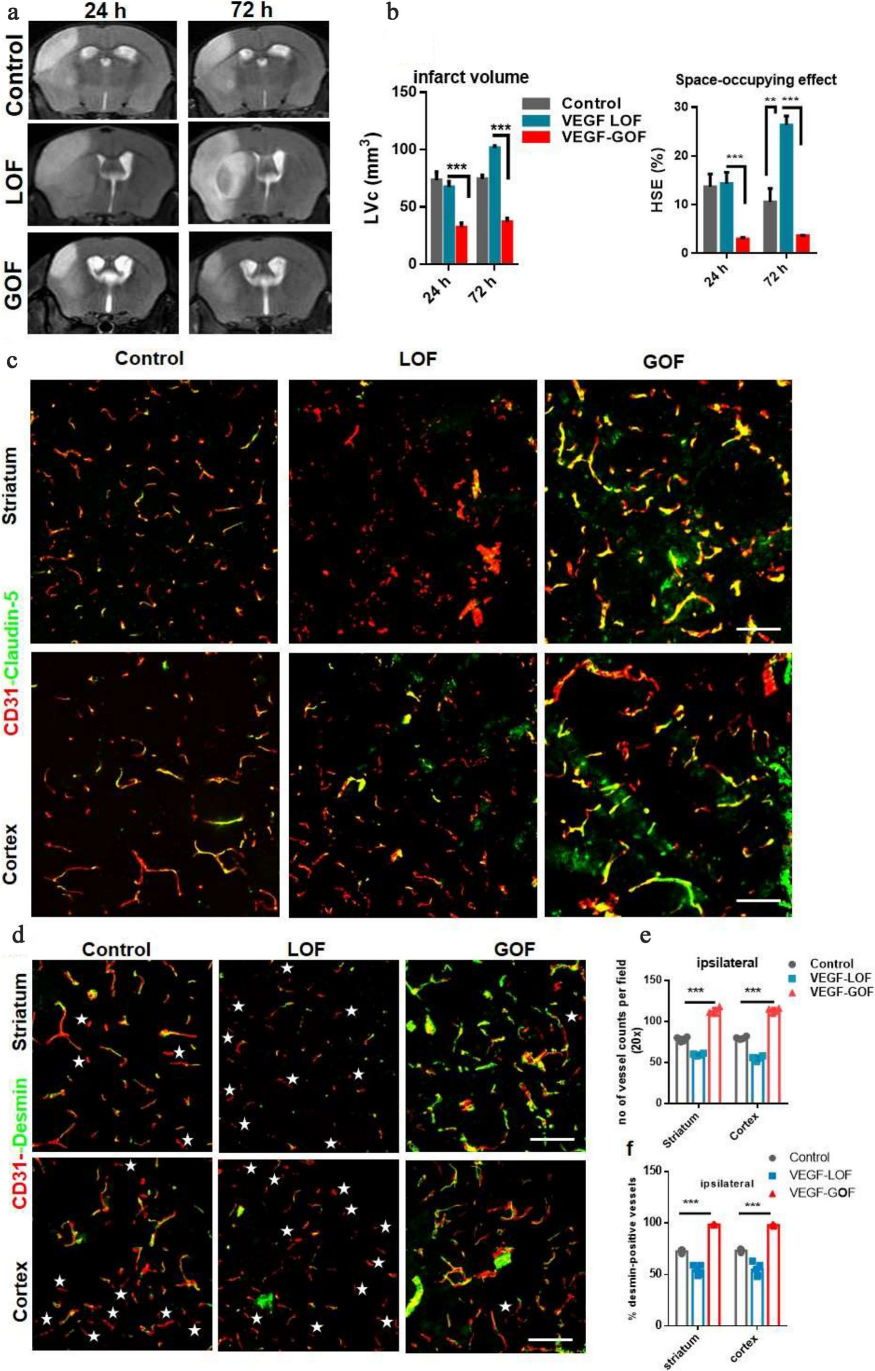
VEGF-induced blood–brain barrier repair in aged animals. (a) Cerebral lesion region is determined by using T2-weighted magnetic resonance images (MRI), representative MRI from control, VEGF-LOF and VEGF-GOF animal 24 h and 72 h post-stroke. (b) Infarct size is significantly reduced in VEGF-GOF already from 24 h post-stroke, whereas VEGF-LOF show a significant increase in the infarct volume 24 h post-stroke and the infarct size further increase 72 h post-stroke. The space-occupying effect in VEGF-GOF is significantly reduced after 24 h post-stroke indicating a decrease in the volume of brain swelling, on the other hand blocking VEGF signaling aggravated brain swelling. *n* = 5 animals per experimental groups. P values were determined by ANOVA (**P < 0.01, ***P < 0.001). Values are represented as ± SEM. (c) Tight junction protein claudin-5 showed increased blood vessel coverage in VEGF-GOF animals, whereas blood vessels in VEGF-LOF and control animals have lower claudin-5 coverage 72 h post-stroke. (d) Highly increased endothelial-pericyte association already 72 h post-stroke is observed in the VEGF-GOF when compared to the VEGF-LOF and control, stars shape marks the microvessels without the pericyte coverage. Magnification 200 × ; scale bar 50 μm. (e) Quantification analysis shows that number of vessels are significantly reduced in the VEGF-LOF and also in control animals when compared to VEGF-GOF animals. (f) quantification of desmin-positive vessels shows only 50% and 65% of, desmin-covered vessels in the VEGF-LOF and control, respectively. Whereas in VEGF-GOF 99% vessels are desmin-positive, *P* values were determined by ANOVA (***P < 0.001). Values are represented as ± SEM. *n* = 10 animals per experimental groups. Magnification 200 × ; Scale bar 20 μm

So far, we performed experiments in young animals. However, stroke is a disease of the aged population and it remains unclear whether VEGF has the same effects on aged brain vasculature. Hence, we studied the effects of VEGF modulation in aged animals (VEGF-GOF/LOF animals, 16–20 months of age). Like in young animals, aged VEGF-GOF resulted in reduced infarction areas with less space-occupying effect due to reduced brain swelling compared to control (Fig. 4a, b). In consistency with these results, the Bederson score assessing the neurological function of animals was significantly improved in the VEGF-GOF at 72 h post-stroke compared to VEGF-LOF and control (Supplementary Fig. 7a). VEGF-GOF also regained the body weight within 72 h (Supplementary Fig. 7b). Furthermore, stroke and brain swelling were aggravated in aged VEGF-LOF compared to controls. Double staining for CD31 and claudin-5 and occludin revealed that VEGF overexpression leads to an increase of claudin-5 and occludin expression and remains unaltered 72 h after ischemic attack (Fig. 4c and Supplementary Fig. 7c). On the contrary, aged VEGF-LOF led to a significantly decreased claudin-5 expression 72 h after stroke, thus disrupting the blood–brain barrier integrity (Fig. 4c and Supplementary Fig. 7c). In addition, pericyte coverage of blood vessels was decreased in aged VEGF-LOF. In aged VEGF-GOF animals, blood vessels were intact and fully covered by pericytes 72 h after stroke (Fig. 4d) (these results are further confirmed with NG2 another pericyte marker (Supplementary Fig. 7d)). Quantification data confirmed that the number of blood vessels in the ipsilateral hemisphere was significantly increased in aged VEGF-GOF and that 99% of the vessels were desmin-positive. However, only 50% of the vessels in aged VEGF-LOF animals and 75% of the VEGF-control vessels were covered by pericytes in the ipsilateral hemisphere (Fig. 4e, f).

Thus, the role of VEGF-GOF in mediating vascular integrity and BBB repair has been demonstrated for both ages. VEGF induction might repair vascular damage after stroke even in elderly mouse.

### Vasculoprotective Effect of VEGF Persists Following Withdrawal of VEGF Transgene Expression

**Fig. 5:**
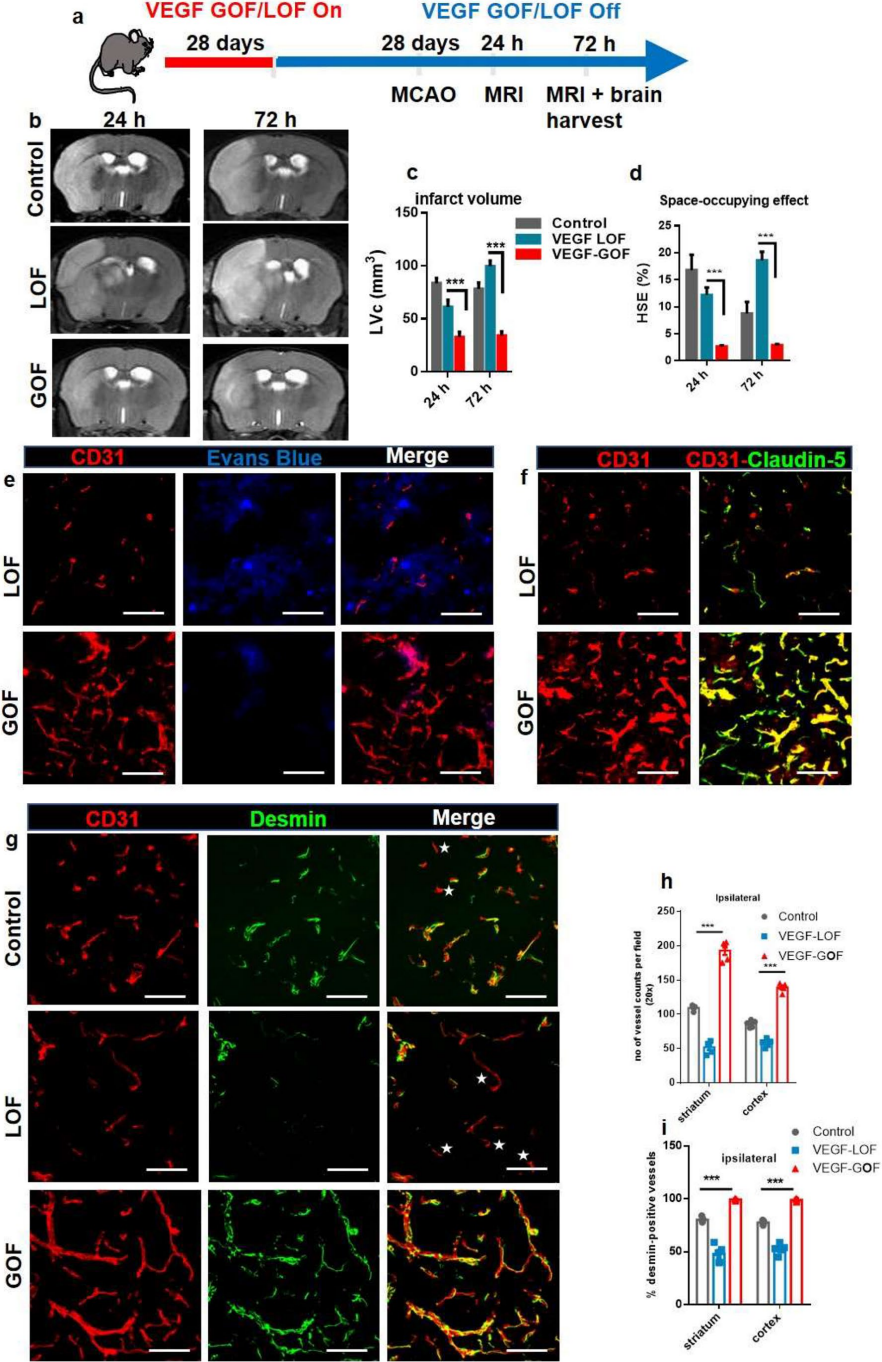
VEGF-induced vascular stability is not reversible after VEGF withdrawal (VEGF on > off). (a) Schematic timeline representation of the experimental procedure. Transgenic expression of VEGF or I-sVEGF-R1 was switched on for 28 days, and then turned off for 28 days before inducing 60 min MCAO. (b) T2-weighted magnetic resonance images (MRI), representative MRI from control, VEGF-LOF (on > off) and VEGF-GOF (on > off) animal 24 h and 72 h post-stroke. (c, d) quantitation of the lesion volume corrected (LVc, mm3) and space-occupying effect attributable to edema (% HSE) after stroke. Infarct size is significantly reduced in VEGF-GOF (on > off) already from 24 h post-stroke, whereas VEGF-LOF (on > off) show a significant increase in the infarct volume 24 h post-stroke and the infarct size further increase 72 h post-stroke. The space-occupying effect in VEGF-GOF (on > off) is significantly reduced 24 h post-stroke indicating a decrease in the volume of brain swelling, on the other hand blocking VEGF signaling (LOF on > off) aggravated brain swelling. *n* = 5 animals per experimental groups. P values were determined by ANOVA (***P < 0.001). Values are represented as ± SEM. (e) Confocal analysis of Evans Blue and CD31 showing an increased in cerebrovascular permeability in the ischemic core in VEGF-LOF (on > off) versus VEGF-GOF (on > off). *n* = 5 in both experimental groups. (f) Confocal images of CD31-claudin-5 showed an increase in the tight junction protein claudin-5 expression in VEGF-GOF (on > off), whereas blood vessels in VEGF-LOF (on > off) have lower claudin-5 coverage 72 h post-stroke. (g) Confocal images show an increase in pericyte coverage of cerebral vessel in VEGF-GOF (on > off) compared with VEGF-LOF (on > off) and control. Star shape marks the microvessels without the pericyte coverage. (h, i) Total number of vessels were increased in the VEGF-GOF (on > off animals) and quantification of desmin-positive vessels show that all the blood vessels are desmin-covered. VEGF-LOF (on > off) showed significant decrease in the number of vessels and the desmin-positive vessels were significantly decreases 72 h when compared to the control and VEGF-GOF (on > off). *n* = 5 animals per experimental groups. *P* values were determined by ANOVA (***P < 0.001). Values are represented as ± SEM. Scale bar 20 μm

To determine whether the vascular stability requires continuous VEGF expression, VEGF was switched off 28 days after induction (i.e., VEGF on > off). After 28 days, animals were subjected to MCA occlusion (Fig. 5a). Results were highly similar to the pre-stroke VEGF condition (without switching VEGF off). T2-weighted imaging demonstrated that VEGF-GOF on > off animals exhibit a significantly reduced infarct volume with a significantly smaller space-occupying effect due to reduced brain swelling and a decreased corrected lesion volume within 72 h post-stroke compared to VEGF-LOF on > off animals (Fig. 5b-d). This might be explained by a significantly increased microvascular density prior to stroke (Supplementary Fig. 8a). VEGF activation over 28 days led to an increased microvessel density which persisted even 28 days after VEGF withdrawal (Supplementary Fig. 8a). Withdrawal of VEGF in GOF on > off had no impact on the blood–brain barrier; it remained vasculoprotective after stroke as shown by Evans Blue experiments (Fig. 5e). In addition, blood vessels in VEGF-GOF on > off animals were vastly covered with claudin-5 demonstrating the stable blood–brain barrier (Fig. 5f). On the contrary, enhanced Evans Blue extravasation revealed the damaged blood–brain barrier in the VEGF-LOF on > off, and immunostainings for claudin-5 revealed the ischemia-induced vascular damage in the VEGF-LOF on > off animals (Fig. 5e, f). Microscopic and quantitative analysis of the vasculature revealed unstable blood vessels in VEGF-LOF on > off animals, whereas the vasculature in VEGF-GOF on > off stayed intact after the ischemic attack (Fig. 5g-i). In addition, we showed that VEGF-GOF on > off exhibit a more rapid angiogenic response than VEGF-LOF on > off as shown by Ki-67-CD31 co-staining (Supplementary Fig. 8b). Under on > off baseline condition, no endothelial cell proliferation was observed, which is contrary to the VEGF-on baseline condition showing that continuous VEGF activation keeps angiogenesis active.

With these studies, we demonstrate two beneficial effects of VEGF activation. Firstly, it provides neuroprotection by containing stroke size, and secondly, VEGF acts as a potent vasculoprotector following stroke. These results indicate that continuous VEGF signaling is not necessary to prevent ischemia-induced damage; however, this aspect is rather attributed to VEGF-induced stablilization of microvasculature.

### Simultaneous VEGF Induction After Stroke Stimulates Early Vascular Repair by Promoting Pericyte Coverage of Microvasculature

**Fig. 6:**
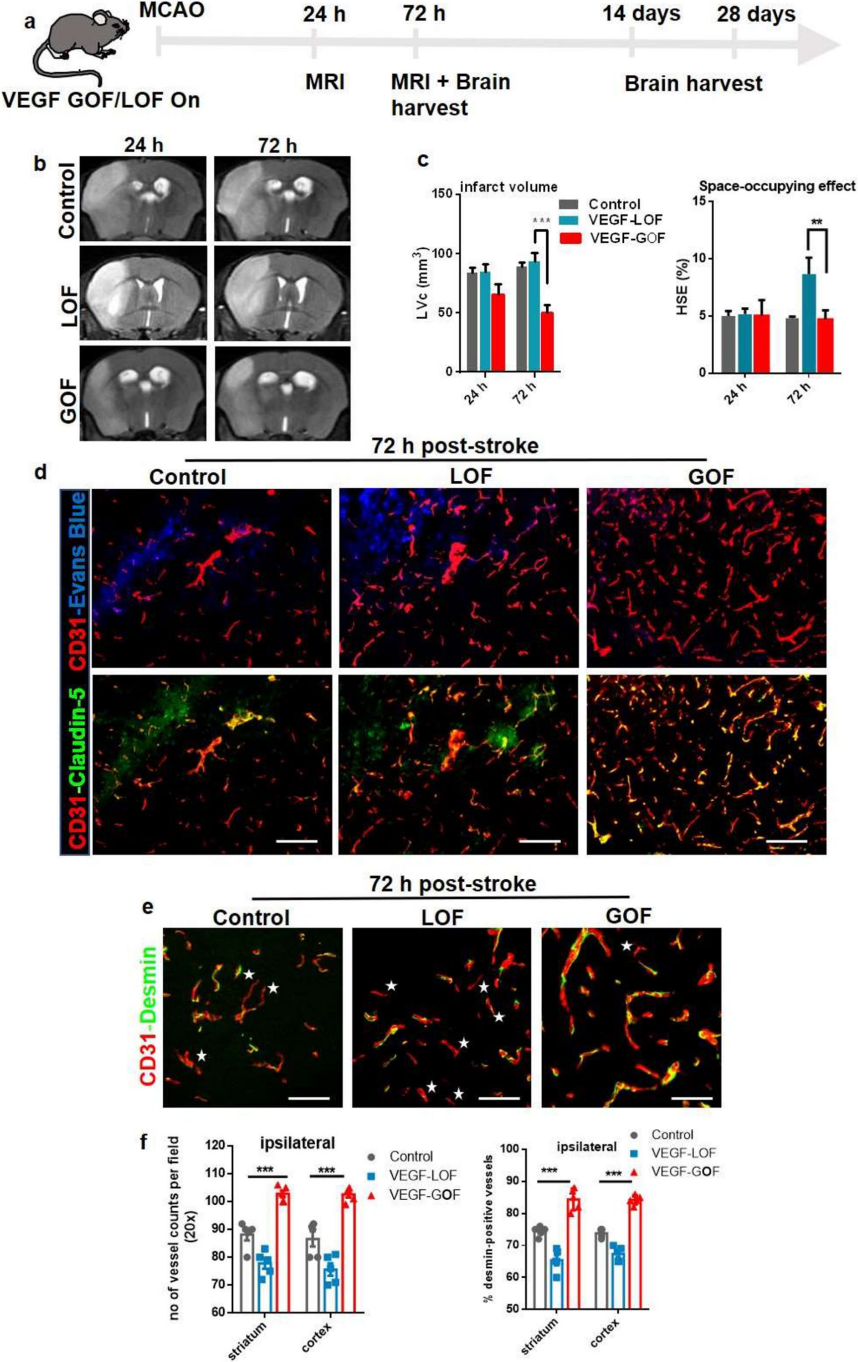
Early VEGF upregulation after stroke contains edema formation and reduces infarct size. (a) Schematic timeline representation of the experimental procedure. (b) T2-weighted magnetic resonance images (MRI), representative MRI from control, VEGF-LOF and VEGF-GOF animal 24 h and 72 h post-stroke. (c) Infarct size is significantly reduced in VEGF-GOF 72 h post-stroke, whereas VEGF-LOF indicate an increase in the infarct size. VEGF-LOF show a significant increase in the space-occupying effect 72 h post-stroke indicating an increase in the volume of brain swelling. *n* = 10 animals per experimental groups. P values were determined by ANOVA (**P < 0.01, ***P < 0.001). Values are represented as ± SEM. (d) Tight junction protein claudin-5 showed increased blood vessel coverage in VEGF-GOF animals and reduced Evans Blue extravasation, whereas blood vessels in VEGF-LOF and control animals have lower claudin-5 coverage and higher Evans Blue extravasation 72 h post-stroke. Magnification 200 × ; scale bar 50 μm. (e) Confocal images of CD31/desmin co-staining show a stable pericyte coverage of blood vessel in VEGF-GOF animals compared with VEGF-LOF and control 72 h post-stroke. Star shape marks the microvessels without the pericyte coverage. Scale bar 20 μm. (f) Quantification data shows a significant decrease in the number of desmin-positive vessels in the ipsilateral hemisphere of the VEGF-LOF 72 h when compared to the control and VEGF-GOF. 90% of the VEGF-GOF vessels in the ipsilateral hemisphere are desmin-positive 72 h after stroke. *n* = 10 animals per experimental groups. *P* values were determined by ANOVA (***P < 0.001). Values are represented as ± SEM

Thus far, we studied the role of pre-stroke VEGF activation in maintaining vascular stability, decreasing infarct size, reducing brain swelling, and restoring blood–brain barrier integrity after ischemia. Next, we sought to determine the functional relevance of VEGF activation immediately after stroke. With the goal of investigating the feasibility of the VEGF signaling system as a potential neuroprotective strategy in the clinical setting, therefore, we activated or blocked VEGF signaling while simultaneously inducing ischemic stroke (Fig. 6a). Development of brain swelling 24 and 72 h after reperfusion was demonstrated by T2-weighted magnetic resonance imaging (Fig. 6b). We demonstrate that simultaneous VEGF onset has a measurable effect on infarct size after 24 h post-stroke in VEGF-GOF. The infarct size was significantly smaller after 24 h, and the corrected lesion was further decreased 72 h post-stroke in the VEGF overexpression animals (Fig. 6c, left). In particular, a smaller space-occupying effect due to reduced brain swelling was observed in VEGF-GOF animals (Fig. 6c, right). Triple staining for CD31, claudin-5, and Evans Blue revealed reduced vascular permeability and increased claudin-5 coverage in VEGF-GOF compared to VEGF-LOF and control 72 h after ischemia. (Fig. 6d). In VEGF-LOF, the increase in brain swelling and high Evans Blue leakage was attributed to loss of vascular integrity. Analysis of confocal images of CD31-staining revealed a reduced pericyte coverage of endothelium in VEGF-LOF animals after 72 h post-stroke (Fig. 6e). Quantification data showed that VEGF-GOF had significantly increased pericyte coverage compared to VEGF-LOF and control 72 h post-stroke (Fig. 6f).

With this experimental paradigm, we show that overexpressing VEGF at stroke onset accelerates early vascular repair mechanisms.

**Fig. 7:**
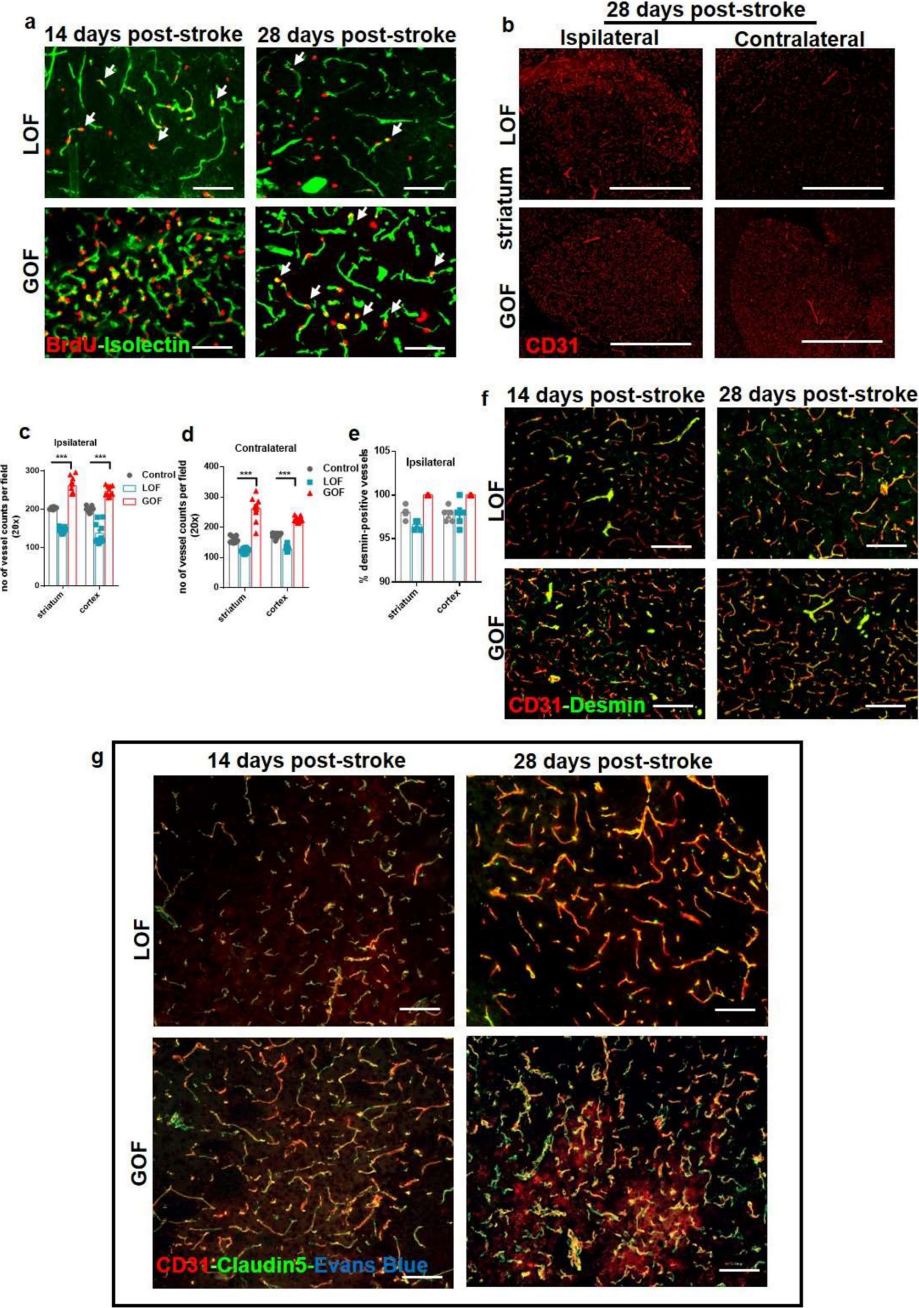
VEGF-induced long-term changes in the late phase of stroke. (a) BrdU labelled cells co-stained with neovascular marker isolectin, showed increased EC proliferation in the VEGF-GOF animals 14 days and 28 days post-stroke. VEGF-LOF animals showed fewer EC proliferation on day 14 and 28 after stroke. (b-d) CD31 staining and quantification data show an increase in the number of blood vessels in the both ipsilateral and contralateral hemisphere of the VEGF-GOF 28 days post-stroke, whereas VEGF-LOF show post-stroke neovascularization only in the ipsilateral region (Scale bar 100 μm). P values were determined by ANOVA (***P < 0.001). Values are represented as ± SEM. (e, f) CD31/desmin co-staining on cryofixed sections and quantification of desmin-positive vessels show no difference in percentage of stable vessels in VEGF-GOF, VEGF-LOF and control. n = 10 animals per experimental groups. P values were determined by ANOVA. Values are represented as ± SEM. (g) CD31/claudin-5 co-staining on cryofixed sections showed no difference in tight junction protein coverage in VEGF-GOF and VEGF-LOF 14- and 28-day post-stroke. No Evans Blue extravasation was observed at these timepoints. Scale bar 50 μm

Next, we aimed to investigate VEGF-induced long-term changes in the late phase of stroke. During the early repair phase, cell recruitment, migration, and vascular sprouting occur, whereas during the late phase of stroke, the vascular system stabilizes, and rehabilitation processes are active. Fourteen days until 28 days after stroke is considered the late phase of stroke; therefore, we studied these two time points. VEGF-GOF exhibited immense angiogenic activity. The EC turnover was high in both ipsilateral and contralateral hemispheres 14 days post-stroke and continued to have enhanced proliferation even 28 days after stroke. The vast endothelial cell proliferation turned the ipsilateral and contralateral vascular system of VEGF-GOF into highly plastic vascular system with a significant increase in the number of the blood vessels (Fig. 7a-d). In comparison, active endothelial cell (EC) turnover in VEGF-LOF was exceptionally low after 14- and 28-day post-stroke as shown by BrdU-Isolectin co-staining (Fig. 7a). Unlike VEGF-GOF, an increased number of vessels in VEGF-LOF were only detected in the ischemic ipsilateral hemisphere and not in the contralateral hemisphere (Fig. 7b-d). Although the number of vessels was lower in the VEGF-LOF animals, 98% were fully covered with the pericytes identical to control and VEGF-GOF animals (Fig. 7e). Co-staining of CD31-desmin showed no difference in pericyte coverage between VEGF-LOF and VEGF-GOF at day 14 and day 28 after stroke (Fig. 7f and Supplementary Fig. 9 showing NG2 marker for pericytes). Triple staining for CD31, claudin-5, and Evans Blue showed no Evans Blue extravasation in both VEGF-GOF and VEGF-LOF groups. Furthermore, no assessable difference was observed in claudin-5 coverage in VEGF-GOF and VEGF-LOF 14- and 28-day post-stroke (Fig. 7g).

Altogether, our data show that activating VEGF signaling parallel to stroke onset can accelerate vascular repair and enhance blood–brain barrier integrity, whereas inhibiting the VEGF signaling intensifies the brain swelling and hinders the vascular repair by reducing the endothelial-pericyte interaction. In response to ischemia, the brain reorganizes and repairs the vascular system itself, even in VEGF-LOF animals.

## Discussion

It is generally recognized that enhanced cerebral edema after stroke is due to blood–brain barrier disruption. Loss of pericytic cells covering the blood vessels can trigger the vascular damage which contributes to the instability of blood vessels and increases vessel permeability [35–37]. In this study, we focused on the role of VEGF in cerebral edema formation and blood–brain barrier (BBB) repair after stroke. Using a unique transgenic research tool allowed for conditional transgenic manipulations of VEGF in the brain, suitable for VEGF gain-of-function (GOF) or, for VEGF loss-of-function (LOF), both in a reversible manner [30], we demonstrated that overexpressing VEGF prior to stroke protects the brain from stroke-induced damages. Blood–brain barrier stayed intact during the sub-acute phase of stroke when VEGF signaling was activated. On the contrary, blocking VEGF signaling prior to stroke had a devastating effect on vascular recovery and BBB stability.

Ischemic preconditioning is a procedure that applies brief, no-lethal, lower threshold ischemic events to the tissue. Preconditioning is performed to develop a resistance and to protect the tissue from subsequent injury [38]. Previous studies show that VEGF is one of the key players in preconditioning-induced neurovascular protection [39–41]. In healthy adult brains, VEGF is expressed at low levels to maintain the normal vasculature [42]. However, after ischemic preconditioning, VEGF expression is upregulated and involved in angiogenic processes [43–45]. Here, we show that VEGF pretreatment without any ischemic preconditioning is more effective in protecting the brain after ischemic stroke. Twenty-eight days prior to the stroke VEGF was induced in VEGF GOF system which evoked a substantial angiogenic response. VEGF-induced endothelial proliferation in cortical, striatal, and hippocampal regions which led to the formation of functional and well-integrated blood vessels. The VEGF-induced neovascularization stabilizes the vascular system prior to the ischemic injury and reduces cerebral infarction after stroke. This is in line with previous findings showing that VEGF induces blood vessels, and enhances cerebral blood flow (CBF), stabilizes cerebral energy state, and reduces cerebral infraction [46]. Furthermore, we show that the newly formed vessels were fully covered with perivascular cells and showed no leakage. Besides inducing angiogenesis, VEGF also contributes to recruiting pericytes and enhancing the expression of tight junction proteins (TJPs), thereby protecting the blood–brain barrier from stroke-related damage. This protective role of VEGF on the blood–brain barrier is not only limited to young animals, but also we were able to verify its protective role in aged animals. Due to our unique genetic system, we for the first time had the possibility to manipulate VEGF expression in terms of LOF and GOF in a well-controlled, conditional manner. We show that activating VEGF signaling simultaneous to the stroke onset can directly impact vascular repair mechanism. In parallel, using our VEGF on/off experimental paradigm, we demonstrate that VEGF can likewise contribute indirectly via vessel formation. VEGF pretreatment for 28 days resulted in fully functional vessels, which persisted even after VEGF withdrawal and enabled the brain to protect itself from subsequent cerebral ischemia. On the contrary, blocking the VEGF signaling pathway (LOF) prior to stroke enhances the brain injury via increasing brain swelling and blood–brain barrier disruption. Based on these observations, we further propose that VEGF simultaneously activates two different pathways to strengthen the vasculature (Fig. 3E). VEGF activation upregulates VEGFR2 and ANG2/Tie2 axis; these molecules have been shown to be involved in sprouting angiogenesis [47–49]. These VEGF signaling pathways turn the vascular system into highly plastic network with enhanced neovascularization. Simultaneously, VEGF upregulates ephrinB2 and PDGF-BB which are involved in pericytes recruitment and enhance the endothelial-pericyte interaction [26, 50]. Overall, our data suggests that Ang2/Tie2 signals make the system highly plastic, constantly forming new vessels in the presence of VEGF, and ephrinB2 downstream signaling pathway actively recruits the pericytes and stabilizes the newly formed vessels. VEGF pretreatment activates these pathways prior to stroke, and the activation further elevates after stroke suggesting their importance in maintaining vascular stability. VEGF pretreatment might be a therapeutic tool in treating high-risk stroke patients.

As mentioned above, one generalization is that stroke-related hypoxia will lead to an upregulation of VEGF, which in turn is responsible for the vascular edema and blood–brain barrier breakdown in the subacute period, i.e., 72 h after stroke. However, in our study, we were able to show that an early increase in VEGF (GOF) expression has a positive effect on infarct volume. Instead of increasing the leakiness and enhancing damage after stroke, VEGF overexpression reduced the infarct volume within 72 h post-stroke, and brain swelling is significantly low. On the contrary, blocking the VEGF signaling pathway (LOF) following stroke enhances the brain injury via increasing brain swelling. Our data suggest that VEGF overexpression within 72 h post-stroke might play a key role in reducing cerebral edema, whereas anti-VEGF therapy at early stages after stroke might augment brain injury. This data is in line with previous studies showing that increasing exogenous levels of VEGF enhance cerebral vascularization and do not increase blood–brain barrier permeability [46]. However, contradictory results were published by Li et al. [51] where pretreatment with VEGF led to intracranial hypertension and aggravated secondary ischemic injury at the early stage of transient middle cerebral artery occlusion (tMCAO). These contradictory results may be due to difference between the timing and method of VEGF manipulation as well as due to the MCA occlusion procedure. In their study, VEGF overexpression was accomplished by injecting recombinant adeno-associated virus 1 (rAAVI)-VEGF165 into the lateral ventrical of adult rats 8 weeks before 2-h transient middle cerebral artery occlusion (tMCAO). In our study, VEGF manipulation was achieved by conditional gain-and loss-of-function mice. Endogenous overexpression of VEGF by conditional mice might influence BBB in a different manner than via rAAVI-VEGF165 injection. Additionally, timing of VEGF pretreatment might play a decisive role in provoking a pro- or anti-permeability effect at the early stage of ischemic stroke. We activated VEGF overexpression 28 days prior to 60-min MCA occlusion.

We used a transient proximal MCAO model; further investigation will be needed to corroborate these findings with permanent focal ischemia model. Currently our group is working on distal permanent MCAO model. In relation to mechanisms, we mainly focused on changes in the junctional proteins claudin-5 and occludin. In addition, it would be interesting to investigate other potential mechanisms of VEGF such as changes in transcytosis and contribution of astrocyte in alternating blood–brain barrier permeability.

Based on the sum of our study, we identify VEGF as an anti-permeability factor and propose that VEGF activation might serve as a therapeutic target in preventing the progression of secondary brain injury by strengthening the vasculature in patients prone to stroke. Furthermore, VEGF administration after stroke may repair the blood–brain barrier and reduces cerebral edema within 72 h after stroke.

## Supplementary Information

Below is the link to the electronic supplementary material.

Supplementary file1 (PDF 2507 KB)

## Data Availability

Not applicable.
