# Supplementary material for: Vascular Endothelial Growth Factor Augments the Tolerance Towards Cerebral Stroke by Enhancing Neurovascular Repair Mechanism
Source: Transl Stroke Res. 2022 Feb 17;13(5):774–91. doi: 10.1007/s12975-022-00991-z (PMC9391249; doi:10.1007/s12975-022-00991-z)
Supplement: Supplementary file 1 — Supplementary file1 (PDF 2507 KB) [file 12975_2022_991_MOESM1_ESM.pdf]

Supplemental Information

Vascular Endothelial Growth Factor augments the tolerance towards cerebral stroke by enhancing neurovascular repair mechanism

Journal: Translational Stroke Research

Adnan Ghori[1], Vincent Prinz[1], Melina Nieminen-Kehlä[1], Simon. H Bayerl[1], Irina Kremenetskaia[1], Jana Riecke[1], Hanna Krechel[1], Thomas Broggini[1], Lea Scherschinski[1], Tamar Licht[2], Eli Keshet[2], Peter Vajkoczy[1]*

1 Department of Neurosurgery, Universitätsmedizin Charité, D-10117 Berlin, Germany.

2 Department of Developmental Biology and Cancer Research, Hebrew University Hadassah Medical School, Jerusalem 91120, Israel

Correspondence to: Peter.Vajkoczy@charite.de

Supplemantary Materials and Methods

Magnetic Resonance Imaging and Analysis

MRI was performed using a 7 Tesla rodent Bruker Pharmascan 70/16 scanner (Bruker Biospin, Ettlingen, Germany) with a 16-cm horizontal bore magnet and a 9 cm (inner diameter) shielded gradient with a H-resonance-frequency of 300 MHz and a maximum gradient strength of 300 mT/m. For imaging a 1H-RF quadratur-volume resonator with an inner diameter of 20 mm was used. Data acquisition and image processing were carried out with the Bruker software Paravision 4.0. During the examinations, mice were placed on a heated circulating water blanket to ensure constant body temperature of 37°C. Anaesthesia was induced with 2.0% and maintained with 1.3-1.8% isoflurane (Forene, Abbot, Wiesbaden, Germany) delivered in a O2/N2O mixture (30/70%) via a facemask under constant ventilation monitoring (Small Animal Monitoring and Gating System, SA Instruments, Stony Brook, New York, USA). For imaging the mouse brain, a T2-weighted 2D turbo spin-echo sequence was used (imaging parameters TR / TE = 4200 / 36 ms, rare factor 8, 4 averages). 20 axial slices with a slice thickness of 0.5 mm, a field of view of (FOV) 2.6 x 2.6 cm and a matrix of 256 x 256 were positioned over the brain from olfactory bulb to cerebellum. Calculation of lesion volume was carried out with the program Analyze 5.0 (AnalyzeDirect, Inc.; Lenexa USA). The hyperintense ischemic areas in T2-weighted images were assigned with a region of interest tool. This enables a threshold-based segmentation by connecting all pixels within a specified threshold range about the selected seed pixel and results in a 3D object map of the whole stroke region. Further, the total volume of the whole object map was automatically calculated.

Real-time PCR

Tissue processing and real-time analysis were performed as described previously [17]. After sacrifice tissues were quickly harvested, snap frozen in liquid nitrogen, and kept at -80°C. Only striatal infarct, peri-infarct and corresponding contralateral striatal region was carefully dissected using mouse brain slicer matrix (Zivic Instruments, Pittsburgh, USA). Total RNA isolation (Invisorb spin tissue RNA kit, Invitek Berlin) reverse transcription (QuantiTect reverse transcription kit, Qiagen), real-time PCR (QuaniTect SYBR green PCR, Qiagen) was performed using company's protocols. Real-time PCR was performed with the 7900 fast real-time PCR System, Applied Biosystems. List of primer used are shown in online supplementary methods. The following primer sequences were used; ephrinB2 forward 5′-AATCACGGTCCAACAAGACG-3′ and reverse 5′-GTCTCCTGCGGTACTTGAGC-3′. GAPDH forward 5′-GGCCTTCCGTGTTCCTACC-3′ and reverse 5′-AACCTGGTCCTCAGTGTAGCC-3′. ANG1 forward 5′- GGATTGAGCTGATGGACTGG and reverse 5′-ACCACCAACCTCCTGTTAGC-3′. ANG2 forward 5′-GATCTTGTCTTGGCCTCAGC-3′ and reverse 5′-GCACTGAGTCGTCGTAGTCG -3′. Tie2 forward 5′-AACAGGATGCTGGAAGAACG-3′ and reverse 5′-ATCACATCTCTTGGCAGTGG-3′. VEGFR2 forward 5′-TGGAGGAAGAGGAAGTGTGC-3′ and reverse 5′-TCAGCTCTTCTGATGCAAGG-3′. PDGF-B forward 5′-GATCCGCTCCTTTGATGATC-3′ and reverse 5′-GTCTCACACTTGCATGCCAG-3′ mVEGF164 forward 5'-AACGATGAAGCCCTGGAGTG-3' and reverse 5'-GACAAACAAATGCTTTCTCCG-3' mVEGF188 forward 5'-AACGATGAAGCCCTGGAGTG-3' and reverse 5'-AACAAGGCTCACAGTGAACG-3'

.

Immunoflourescence and Confocal Analysis

Immunofluorescence and confocal analysis were performed as described previously[17]. The brains were perfused with PBS and immediately shock frozen in chilled isopentane (sigma-aldrich). Immunofluorescence studies were performed on frozen sections 20 µm made on cryostat (HM560 microm GmbH). The sections were fixed in methanol for 10 minutes then incubated with blocking solution containing 1% Casein in PBS for 30 minutes followed by the primary antibodies overnight at 4°C or 2 h at room temperature [rat anti CD31 (BD Pharmingen 550274; 1:50), rabbit anti-desmin (abcam ab15200; 1:100), mouse anti-desmin (Thermo Fischer scientific 1:200), mouse anti-NeuN (MAB377 Millipore; 1:200), rabbit anti-EphB4 (sc-5536 Santa Cruz; 1:100), Goat anti-Ephrin-B2 (GT15026 Neuromics; 1:100), Rabbit anti-Ki-67 (RM-9106-S1 Thermo Fisher scientifics; 1:200), rat anti-KI-67 (Clone TEC-3, Dakocytomation; 1:25), rabbit anti-NG2 (AB5320 Millipore; 1:200), chicken anti-GFAP (abcam ab4674; 1:1000), mouse anti-Claudin-5 (35-2500, invitrogen; 1:100), rabbit anti-ANG-1 (abcam ab8451; 1:100), rabbit anti-ANG-2 (abcam ab8452; 1:100), rabbit anti-Tie2 (C-20) (Santa Cruz sc-324; 1:150), rat anti-CD11b (abcam ab8878; 1:100), rabbit Anti-EphrinB2 (phospho Y316) antibody (abcam ab119323)], after three washes with 0.5% casein in PBS , the sections were incubated with secondary antibody for 2hr [Cy3, FITC, Cy5

and AMCA-conjugated antibodies from Dianova were used ; 1:200 dilution]. Sections were washed with PBS and they processed for double or triple staining.
For imaging, the researcher was blinded to the IF-samples and we used a Carl Zeiss LSM 5 exciter confocal laser-scanning microscope equipped with an argon/neon laser and a Carl Zeiss Axio observer Z1 inverted immunofluorescence microscope equipped with standard DAPI, FITC, Cy3 and Cy5 filter.

Injection and analysis of the Fluorescent Tracer (FITC-dextran)

FITC-Dextran 4kDa (Sigma-Aldrich) was used to evaluate the blood-brain barrier permeability. Animals were administered intravenously with FITC-Dextran (concentration: 25 mg/ml, dissolved in 0.9% saline, 5 mg in 200 µl per animal) to circulate for 2 min. After 2 min the animals were sacrificed, brains were removed and divided into ipsilateral and contralateral hemispheres, weighed, lysed in 500 µl PBS. Homogenized using Dispomix homogenizer and centrifuged at 16 000 rpm at 4°C for 20 min. The supernatants were transferred to a black 96-well plate for fluorescence measurements (Greiner bio one #655209) (100 µl duplicates). Fluorescence measurement using TECAN (infinity 200) was performed at excitation 490 and emission 520 nm. The measurement was converted (µg/0.5ml → µg/ml), based on weight (the measurement results in µg/ml, the number of µg is divided by the weight of the brain/hemisphere). All quantifications were done blinded to the researcher.

BrdU Delivery and BrdU Staining

In order to label the proliferating cells, the DNA duplication marker 5-bromodeoxyuridine (BrdU) was delivered to the immediately after the operation. The BrdU (Sigma) was dissolved in saline and injected intraperitoneally at a timely dose of 0.1mg BrdU/gram of body weight. Optimal formula for an adult mouse is 3mg BrdU in 300 ml. Tissues sections were fixed with methanol for 10 minutes. Then the sections were rinsed twice with PBS. Tissue sections were first heated in a formamide solution for 2 h at 65_C to denature DNA (50% formamide,40% dH2O,10% 20x Saline-Sodium Citrate (SSC: 175.3g NaCl; 88.2g sodium citrate in 800 ml dH2O)). Tissues were then washed once for 5 min in 2x SSC. Next, to break any remaining hydrogen bonds between the nitrogenous bases of nucleic acids, slices were heated at 37°C for 30 min in 2N HCl (2NHCL=17.5ml HCL in 100 ml H2O). To regain pH balance, tissue sections were placed for 10 minutes in a borate buffer (borate buffer = 0.62g boric acid in 100ml H2O and 0.95g sodium tetraborate in 100ml H2O;1:1). Borate buffer was removed and the sections were incubated in blocking solution for 30-60 minutes. 1% casein in PBS was used as a blocking buffer. Next, anti-BrdU antibody in PBS with triton and 10% FBS was added and sections were incubated overnight for 4°C. Next day primary antibody was removed and rinsed with twice with PBS. Then secondary antibody in PBS with triton and 10% FBS was added and incubated at 37°C for at least 1 hour. Next, secondary antibody was removed and rinsed with twice with PBS and then the slides were coversliped.

Neurological score

The test was conducted after the animals were recovered from anaesthesia and again on the day of sacrifice. Mice were held gently by the tail, suspended couple of meters above the floor, and were tested for forelimb flexion, resistance to lateral push and circling behaviour. A grading scale of 0-3 was used to assess the effects of occlusion. Mice with no neurological deficits extended both forelimbs toward the floor, hence were assigned grade 0. Mice with ischemia flexed the forelimb contralateral to the injured hemisphere; grades were assigned from 1-3 depending upon the severity of resistance to the lateral push and circular behaviour. All the experiments were performed in a blinded manner.

Software

Analyze 10.0, Image J, Graphpad Prism (6.01) softwares were used for data analysis and figures.

Supplementary Figures

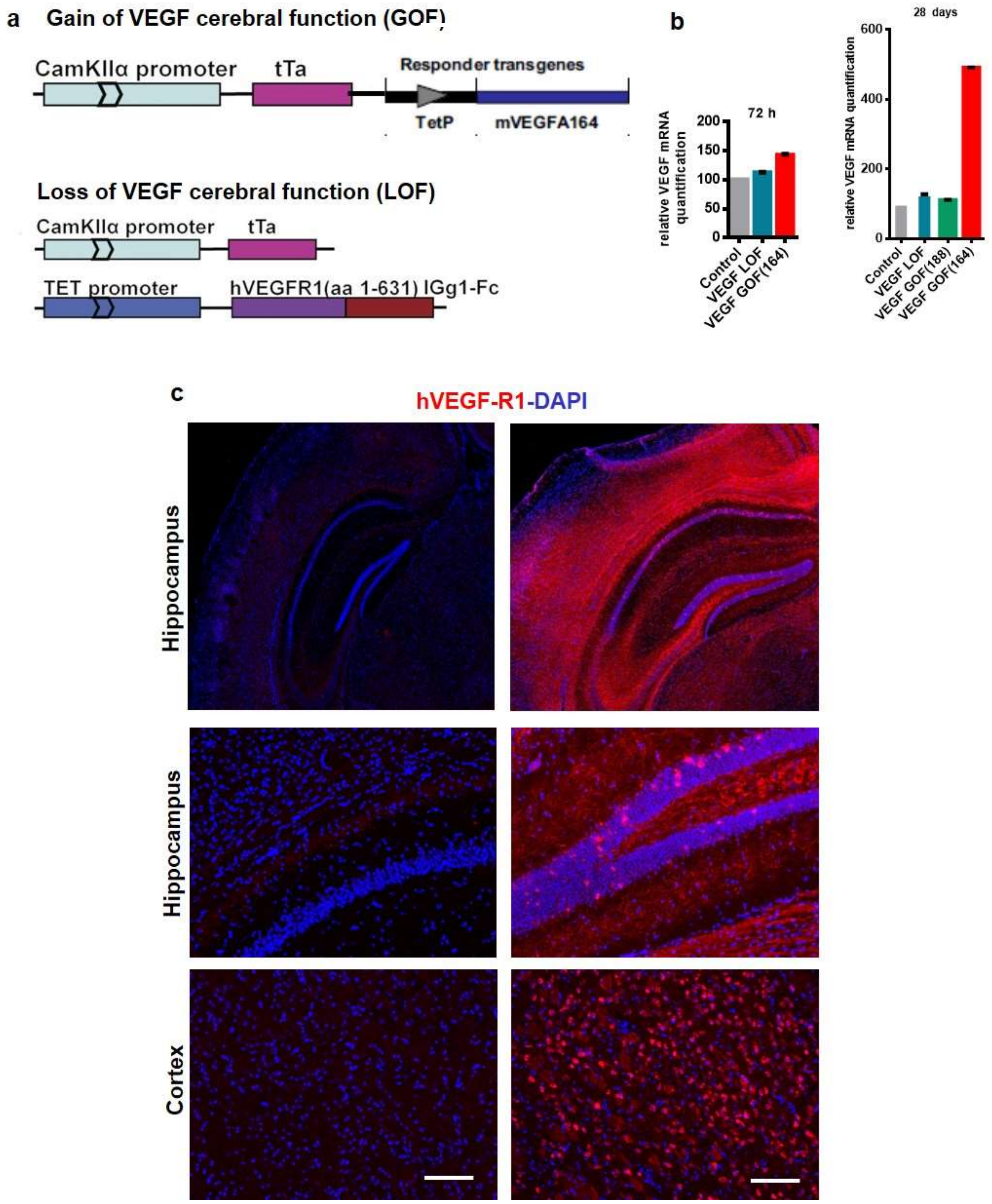

Supplemental Fig. 1 a Schematic representation of VEGF-Gain of function (GOF) and VEGF-Loss and function (LOF). Brain-specific expression is achieved by using a driver transgenic line composed of tet-regulated transactivator protein driven by a CamKIIα promoter. For VEGF gain-of-function (GOF), a tet-responsive VEGF164 transgene was generated and for VEGF loss-of-function (LOF), a tet-responsive VEGF soluble receptor (I-sVEGF-R1, also known as sFlt1) that sequesters VEGF and preclude signaling was generated and employed to reversibly induce and repress VEGF signaling at will. **b** VEGF expression dynamics: Quantification of endogenous and induced VEGF and I-sVEGF-R1 levels. Real-time quantitative PCR of total mRNA at 72 h and 28 days after VEGF manipulation. Relative levels of expression were standardized to control levels. VEGF-gain of function (GOF) show already an increase in its mRNA level 72 h after activation and level increased upto 5x 28 days after

VEGF activation. The VEGF upregulation was only seen for isoform 164 and not for 188. Note that VEGF mRNA level do not significantly change in I-sVEGF-R1–induced mice (LOF), as expected from the posttranslational nature of inhibition, its expression is similar to the control animals. (n=5 for each group, ±SEM). **c** LOF verification: Immunostaining for the extracellular domain of FLT1 in CamKIIα -tTa/TET- I-sVEGF-R1 in LOF animals. Magnification x200; scale bar, 50 μm.

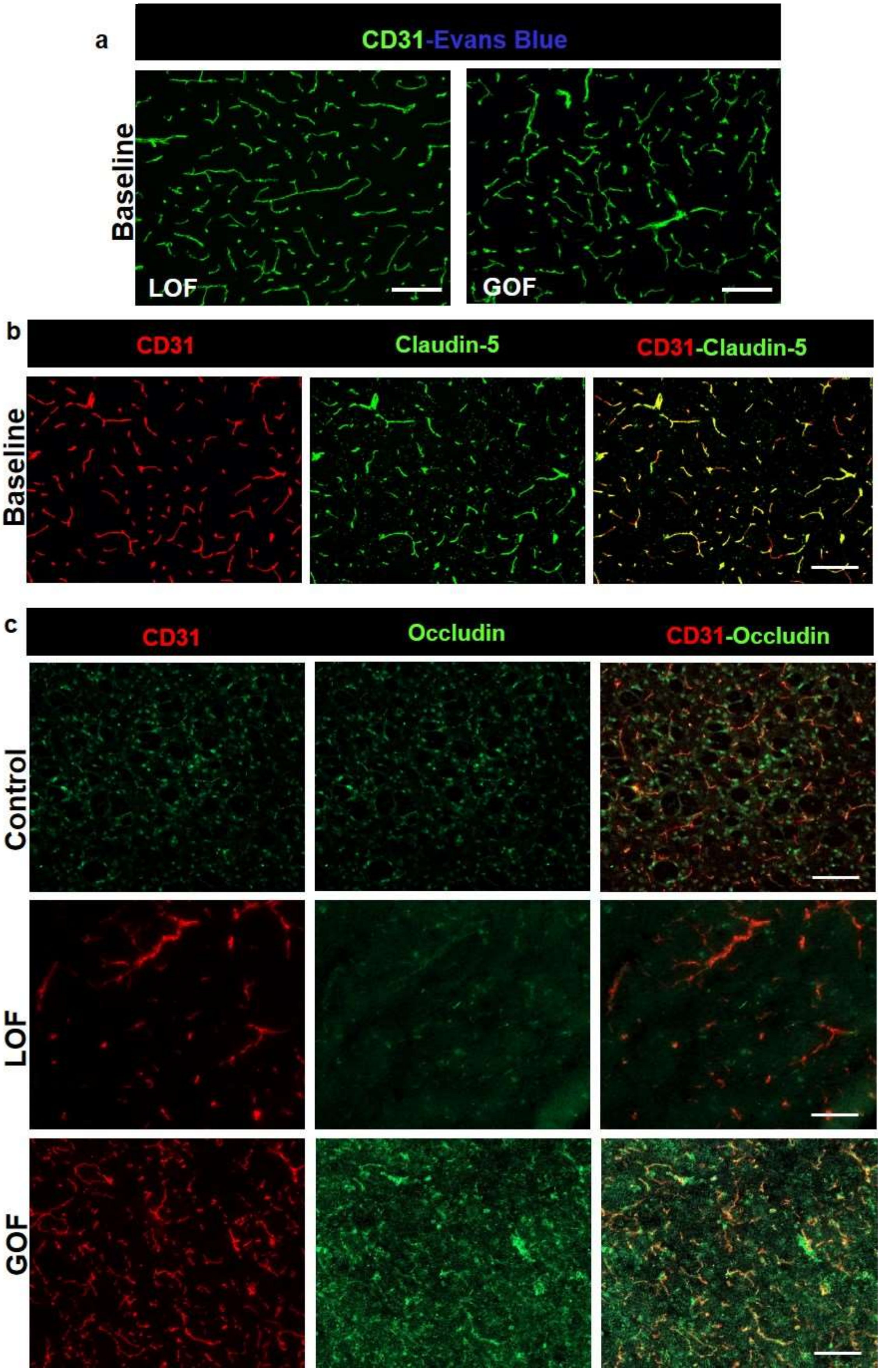

Supplemental Fig. 2 a Baseline study shows no Evans Blue extravasation in both LOF and GOF system. Representative images are taken from striatal region. **b** Expression pattern of tight junction protein claudin-5 in brain under normal condition. **c** Tight junction protein occludin showed increased blood vessel coverage in VEGF-

GOF, whereas blood vessels in VEGF-LOF and control have lower claudin-5 coverage after cerebral ischemia. Magnification x200; scale bar, 50 μm.

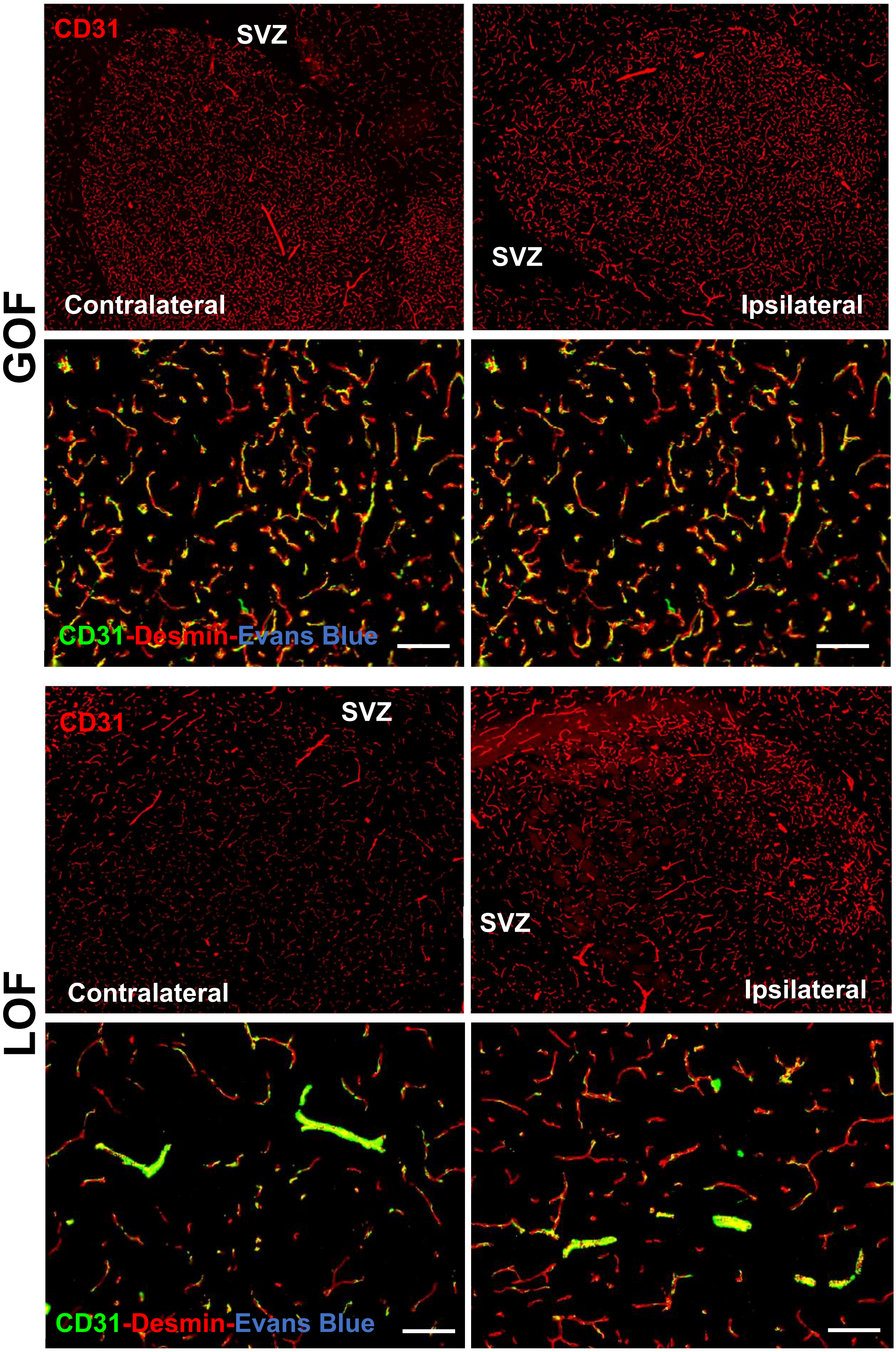

Supplemental Fig. 3 Activating VEGF (GOF) for 28 days lead to an enhanced endothelial cell proliferation. CD31 staining show that number of vessels are increased in both hemispheres. Confocal images of CD31-desmin costaining show that blood vessels are fully covered by the pericytes and are stable in VEGF-GOF. Blocking of

VEGF signaling (LOF) for 28 days did not increase or decrease the number of the vessels. Confocal image of CD31-desmin staining show that vessels are stable and fully covered by the perivascular cells. Magnification x200; scale bar, 50 μm.

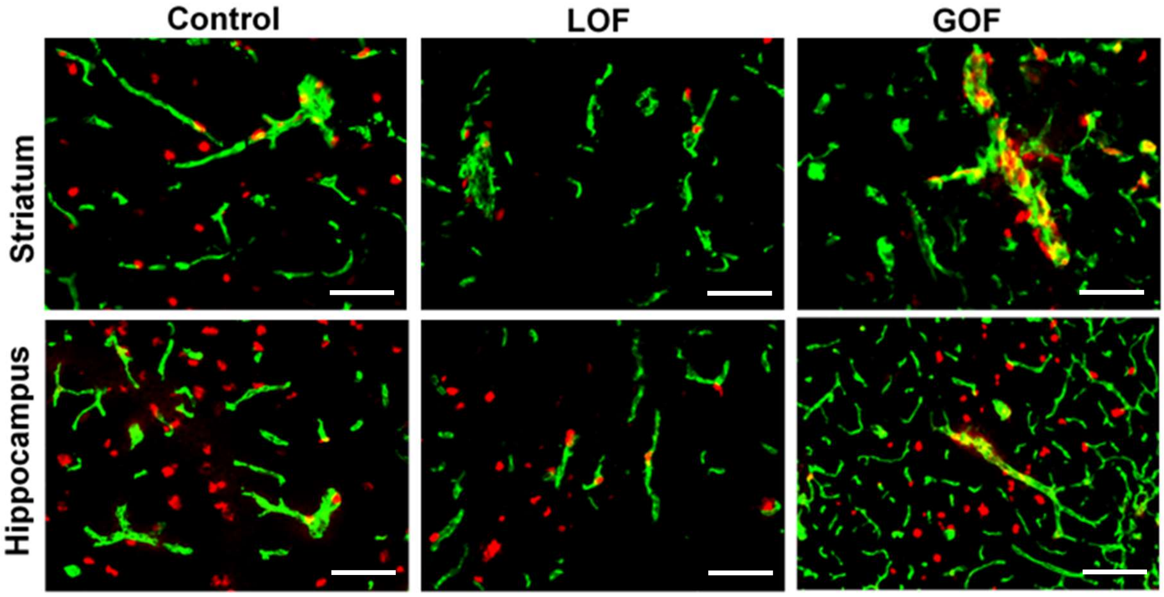

Supplemental Fig. 4 VEGF overexpression (GOF) enhances endothelial cell (EC) proliferation. KI-67 labelled cells co-stained with CD31 showed increased EC proliferation in the VEGF-GOF 72h post-stroke. VEGF-LOF and control group showed fewer proliferating endothelial cells. Representative images are taken 72h after stroke from striatum and hippocampus region. Magnification x200; scale bar, 50 μm.

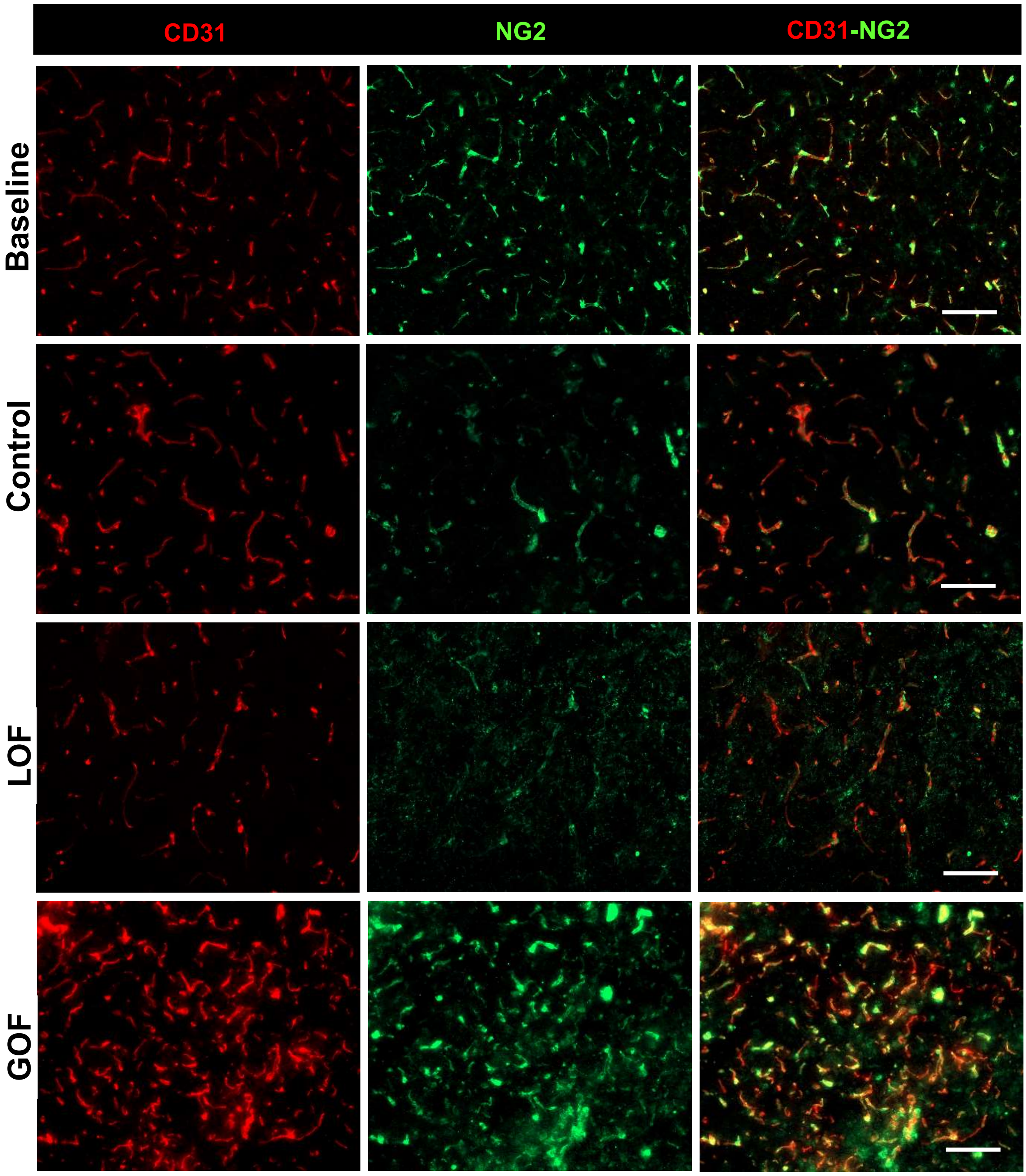

Supplemental Fig. 5 Endothelial-pericyte interaction shown by CD31/NG2 in brain under normal (baseline) condition. CD31/ NG2 co-staining on cryofixed sections shows the enhanced pericyte coverage of cerebral vessel in VEGF-GOF animals compared with LOF and control animals. Magnification x200; scale bar, 50 µm.

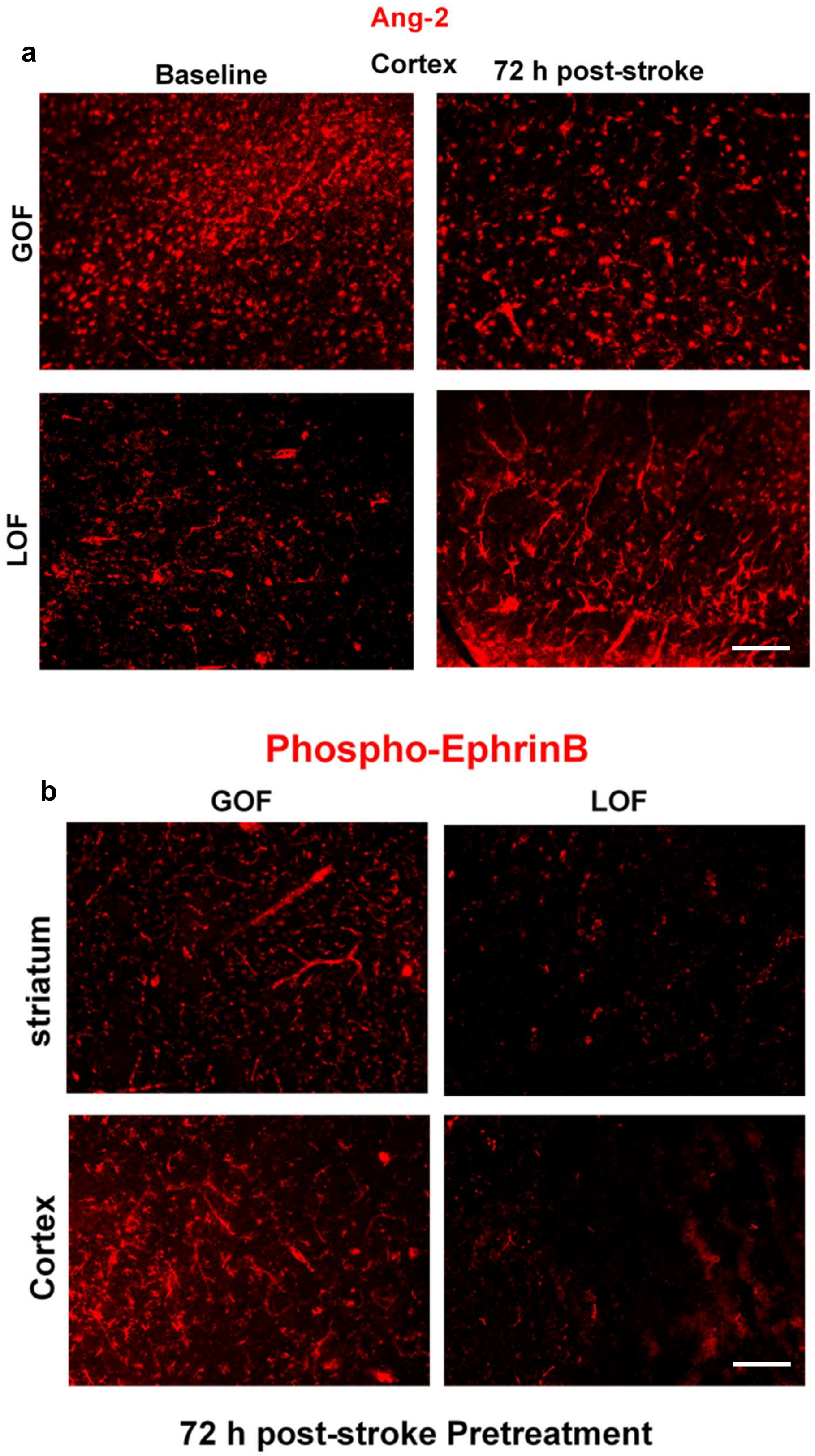

Supplemental Fig. 6 a Confocal images of ANG-2 staining show that an increase in the ANG2 expression in the VEGF-GOF under both baseline and ischemic condition. ANG2 expression in VEGF-LOF only show an upregulation 72 h post-stroke but not under baseline condition. **b** VEGF overexpression (GOF) lead to an increase in ephrinB phosphorylation 72 h post-stroke in striatal and cortical regions of the ipsilateral hemisphere. VEGF-LOF leads to a diminished ephrinB phosphorylation 72 h post-stroke. Magnification x200; scale bar, 50 µm.

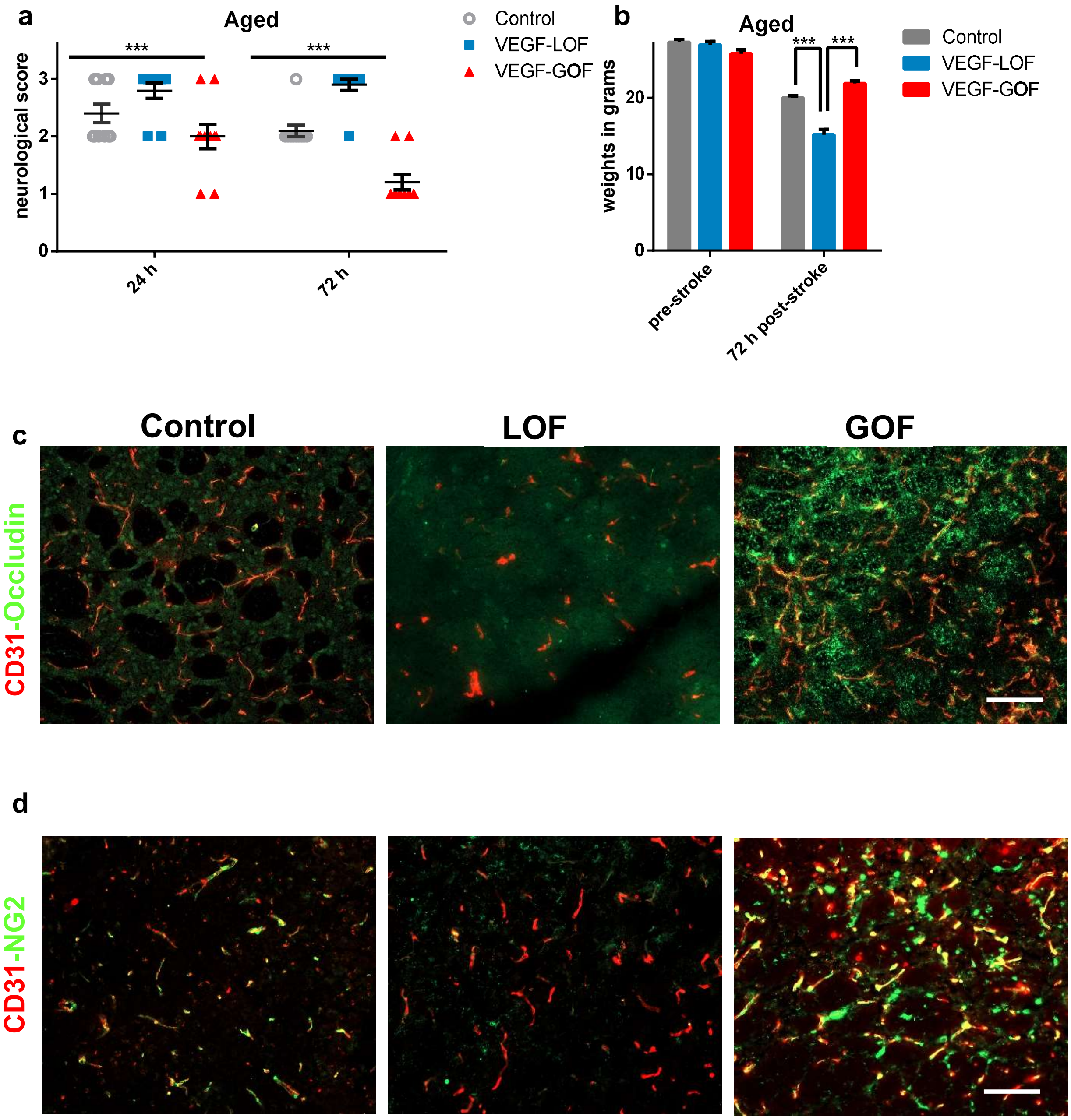

Supplemental Fig. 7 a Neurological function recovers in the VEGF-GOF 72 h post-stroke when compared to the VEGF-LOF and control. **b** VEGF-LOF animal do not gain weight as fast as VEGF-GOF and control animals 72 h post-stroke P values were determined by ANOVA (***P < 0,001). Values are represented as ± SEM. n=10 animals per experimental groups. **c** Tight junction protein occludin showed increased blood vessel coverage in aged VEGF-GOF, whereas blood vessels in aged VEGF-LOF and aged control have lower claudin-5 coverage 72 h after cerebral ischemia. **d** CD31/ NG2 co-staining on cryofixed sections shows the enhanced pericyte coverage of cerebral vessel in aged VEGF-GOF animals compared with aged VEGF-LOF and aged control animals. Magnification x200; scale bar, 50 µm.

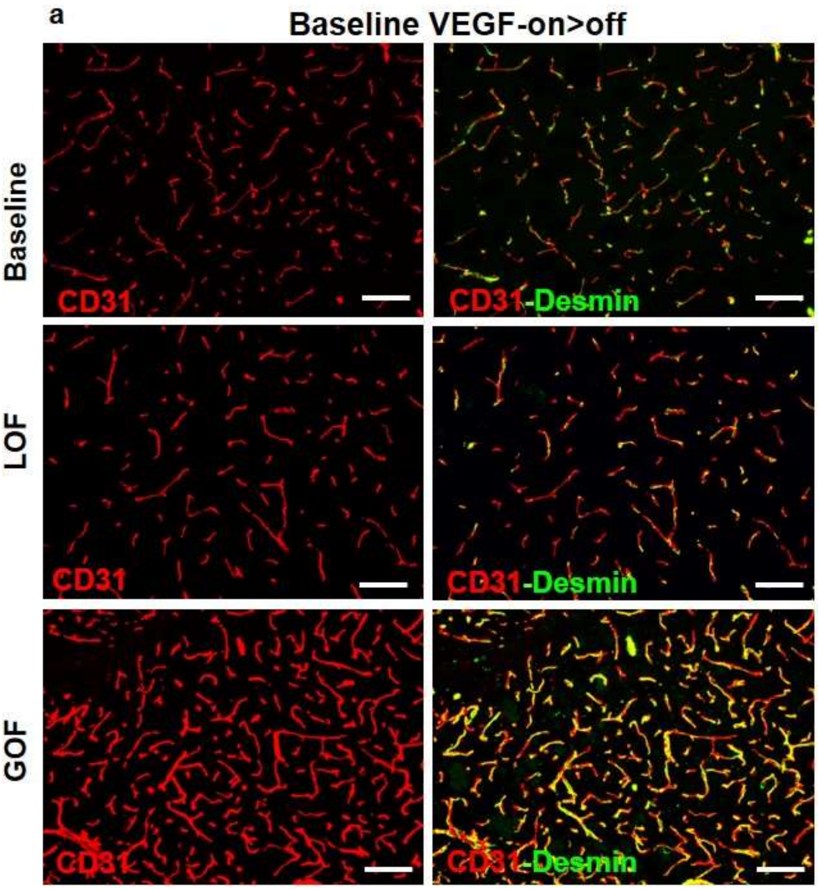

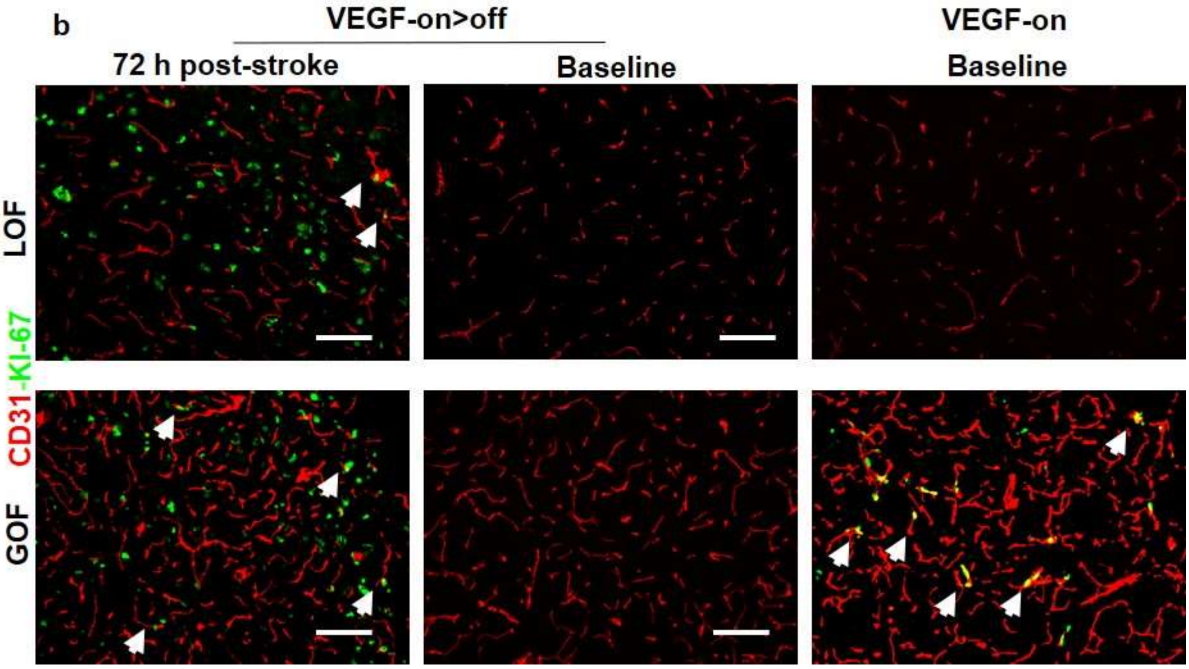

Supplemental Fig. 8 a Confocal images show an increase in the microvascular density in the VEGF overexpression animals (GOF) which persist even 28 days after VEGF withdrawal. VEGF-LOF animals did not show an increase or decrease in the microvessel density after VEGF on>off. **b** Ki-67 labelled post cells co-stained with CD31 showed fewer EC proliferating cells in the VEGF-LOF on>off animals and increased EC proliferating in VEGF-GOF on>off animals 72h post-stroke. Representative images are taken 72h after stroke from striatum region. Under on>off baseline condition no EC proliferation was observed in either of the groups. Under VEGF-

on Baseline, increased EC proliferation is only seen in VEGF activated group (GOF) but not in VEGF-inhibited group (LOF). Magnification x200; scale bar, 50 µm.

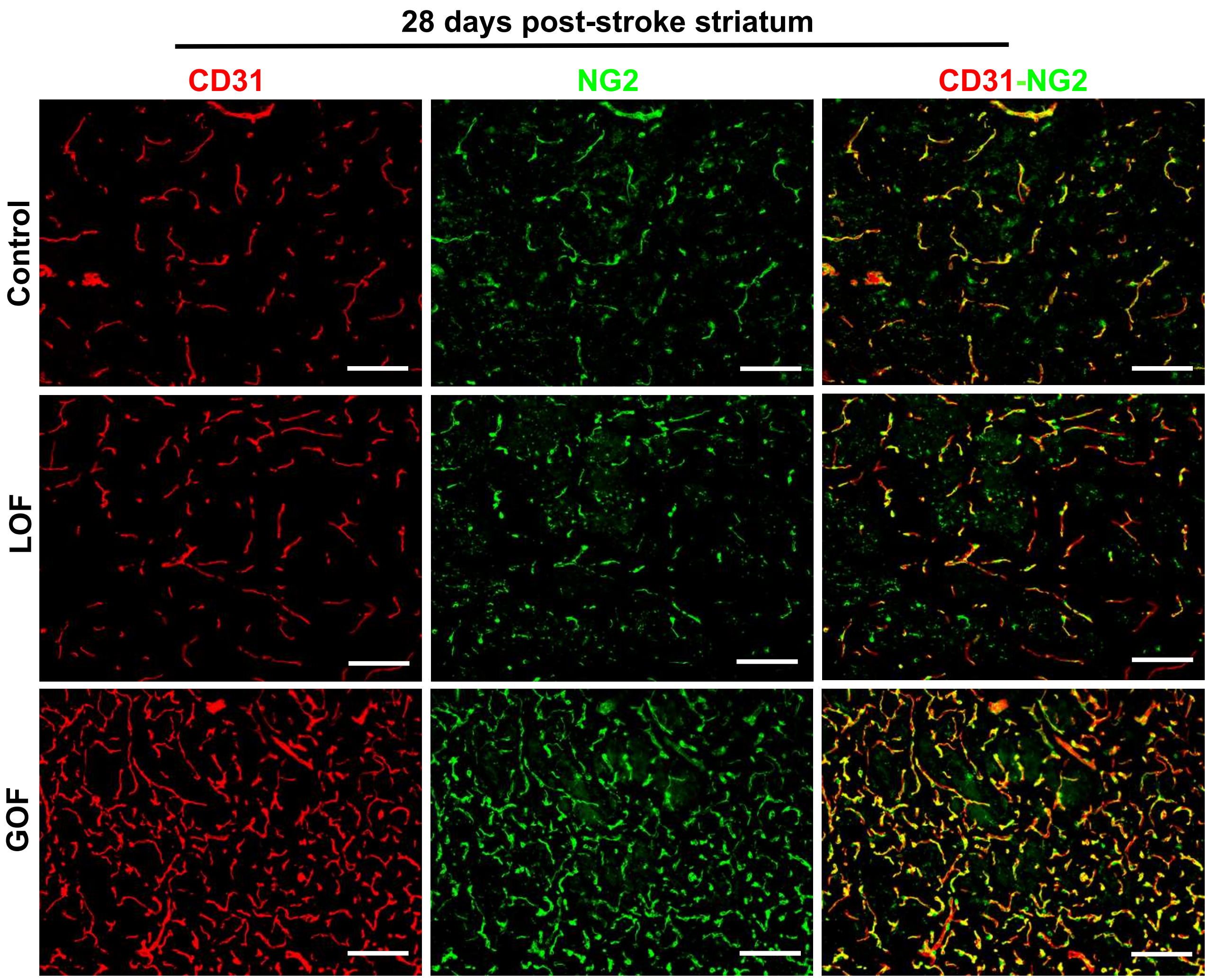

Supplemental Fig. 9 CD31/NG2 costaining on cryofixed sections show that all the vessels are pericyte covered 28 days after stroke in GOF, LOF and control animals. Magnification x200; scale bar, 50 µm.
